# Health Related Quality of Life in a Dutch Rehabilitation Population: Reference Values and the Effect of Physical Activity

**DOI:** 10.1371/journal.pone.0169169

**Published:** 2017-01-06

**Authors:** Leonie A. Krops, Eva A. Jaarsma, Pieter U. Dijkstra, Jan H. B. Geertzen, Rienk Dekker

**Affiliations:** 1 University of Groningen, University Medical Center Groningen, Department of Rehabilitation Medicine, Groningen, The Netherlands; 2 University of Groningen, University Medical Center Groningen, Department of Oral and Maxillofacial Surgery, Groningen, The Netherlands; 3 University of Groningen, University Medical Center Groningen, Center for Sports Medicine, Groningen, The Netherlands; Saint Louis University, UNITED STATES

## Abstract

**Purpose:**

To establish reference values for Health Related Quality of Life (HRQoL) in a Dutch rehabilitation population, and to study effects of patient characteristics, diagnosis and physical activity on HRQoL in this population.

**Method:**

Former rehabilitation patients (3169) were asked to fill in a questionnaire including the Dutch version of the RAND-36. Differences between our rehabilitation patients and Dutch reference values were analyzed (t-tests). Effects of patient characteristics, diagnosis and movement intensity on scores on the subscales of the RAND-36 were analyzed using block wise multiple regression analyses.

**Results:**

In total 1223 patients (39%) returned the questionnaire. HRQoL was significantly poorer in the rehabilitation patients compared to Dutch reference values on all subscales (p<0.001) except for health change (p = 0.197). Longer time between questionnaire and last treatment was associated with a smaller health change (p = 0.035). Higher age negatively affected physical functioning (p<0.001), social functioning (p = 0.004) and health change (p = 0.001). Diagnosis affected outcomes on all subscales except role limitations physical, and mental health (p ranged <0.001 to 0.643). Higher movement intensity was associated with better outcomes on all subscales except for mental health (p ranged <0.001 to 0.190).

**Conclusions:**

HRQoL is poorer in rehabilitation patients compared to Dutch reference values. Physical components of HRQoL are affected by diagnosis. In rehabilitation patients an association between movement intensity and HRQoL was found. For clinical purposes, results of this study can be used as reference values for HRQoL in a rehabilitation setting.

## Introduction

During the past decades, the perspective on health care shifted from mainly biomedical to more biopsychosocial [1]. In the biopsychosocial model, health is described as an interaction between biological, psychological and social aspects [2]. Health Related Quality of Life (HRQoL) is a typical example of a biopsychosocial construct, by its biological (i.e. physical functioning), psychological (i.e. mental health) and social aspects (i.e. social functioning) [3]. Through this shift in health perspective, improving HRQoL tends to become of more importance in present health care. This shift in perspective went simultaneously with an increased demand towards measuring the effectiveness of health care [3]. Taking this together, this highlights the importance of measuring HRQoL in today’s health care [4].

Especially in rehabilitation, improving HRQoL is one of the important goals because of the permanent effects of most impairments. In the treatment of Multiple Sclerosis (MS) patients, HRQoL forms an important consideration [5] since it is highly sensitive to changes in disease status. HRQoL is frequently assessed by using the Short Form– 36 (SF-36), as compiled by the Medical Outcome Study [6]. The SF-36 is highly correlated (0.99) with the RAND-36. Both questionnaires consist of exactly the same 36 items, and only differ slightly in the scoring procedure [7]. Additional to the shared 8 subscales, the RAND-36 has a subscale “health change over the past year”.

In earlier research, quality of life in rehabilitation outpatients proved to be lower compared to the general population [8]. That study used the abbreviated version of the World Health Organization Quality of Life questionnaire, whereby Quality of Life is divided in different subscales compared to the RAND-36 or SF-36. Four studies measured HRQoL using the RAND-36 in a diagnosis group that is represented in the rehabilitation population of the current study [9–12]. Only for people with a lower limb amputation, we found HRQoL as measured by the Dutch translation of the RAND-36 [10,12]. Lower limb amputees scored lower on physical functioning, role limitations–physical and pain compared to control subjects [10]. To the best of our knowledge, besides these four studies, HRQoL was only measured with the SF-36 in different non-Dutch populations, for the diagnoses in the current study [13–39]. Since the SF-36 does not include the health change element, reference values for that element were not present. In general HRQoL was lower in lower limb amputee patients, chronic pain patients, MS patients and spinal cord injured (SCI) patients compared to the general population [9,10,13–15,25,34,38]. Most studies on HRQoL focus on only a small part of the rehabilitation population. However, including various diagnoses of the rehabilitation population allows also a direct comparison of HRQoL between these diagnoses. The aim of the current study is establish reference values for HRQoL in a Dutch rehabilitation population, and to study effects of patient characteristics, diagnosis and physical activity on HRQoL in this population.

## Methods

### Participants

A total of 3169 rehabilitation patients were invited to participate in this study. All of them completed their rehabilitation program in the Center for Rehabilitation of the University Medical Center Groningen, the Netherlands. All rehabilitation patients of 18 years or older, treated between the 1^st^ of January 2009 and 31^st^ December 2011 were invited. Excluded were cardiac or pulmonary rehabilitation patients since they were treated in a different treatment framework, and patients with a diagnosis of orthopedic origin since they were treated mostly monodisciplinary.

### Questionnaire

Participants were asked to fill in a questionnaire including the validated Dutch version of the RAND-36 [40,41] and questions on sports participation [42]. The RAND-36 is a profile based measurement instrument, of which scores on the following 9 subscales are calculated: Physical functioning, Social functioning, Role limitations–physical, Role limitations–emotional, Mental health, Pain, Vitality, General health and Health change.

### Procedure

The patient’s names, addresses, diagnosis, gender, date of birth, and date of last treatment were retrieved from the database of the Center for Rehabilitation of the University Medical Center Groningen. All potential participants received the questionnaire including a cover letter and an informed consent form by post. Potential participants were asked to either fill in and return the paper questionnaire, or fill in the online questionnaire, by using the provided link. After being informed that participation was voluntary and data would be processed anonymously, participants gave their written informed consent. Participants who completed the online questionnaire were asked to return their written informed consent by post. Moreover we assumed that by filling in the questionnaire, the participant declared willingness to participate. Online questionnaires were filled in using the Unipark software (QuestBack GmbH, Berlin, Germany) which fulfills data protection and security requirements (ISO 27001). Prior to sending the questionnaire, all potential participants were coded using a participant number. The online questionnaire was filled in using provided login credentials which were based on the participant number. The paper questionnaire was also coded with the predetermined participant number, whereby no information that can lead to the participant was present on the questionnaire, except for the participant number of which the key was only available to the involved researchers. The study protocol was approved by the Medical Ethical Committee of the University Medical Center Groningen, the Netherlands (METc 2012.450).

### Data analysis

Differences between participants and non-participants were analyzed using independent samples t-tests (age and follow-up period) and chi squared tests (diagnoses and gender). Despite scores on some of the subscales were non-normally distributed (skewness and kurtosis divided by their standard deviation > 1.96), differences between participants and a healthy Dutch reference population [40] for all 9 subscales were analyzed using independent samples t-tests, because of the large sample size. Radar plots were created to elucidate the scores on the subscales for different diagnoses, and for the entire rehabilitation population in comparison to Dutch reference values. Multiple regression analyses were performed to statistically predict scores on the 9 subscales based on follow up (months between last treatment and questionnaire), gender, age, diagnosis and movement intensity. Predictors were entered block wise. Patient characteristics were entered first, diagnosis was entered second, and movement intensity was entered third. When significant effects for movement intensity and for any other predictor were found, interaction effects between these predictors were explored and entered as a fourth block. Movement intensity was calculated by multiplying the activity specific intensity (MET) [43] with the number of hours per week that the activity was performed. Diagnoses were entered using dummy variables, in which MS formed the reference group. Statistical analyses were performed using SPSS 20.1 (IBM, New York). The level of significance was set at p < 0.05.

## Results

**Fig 1:**
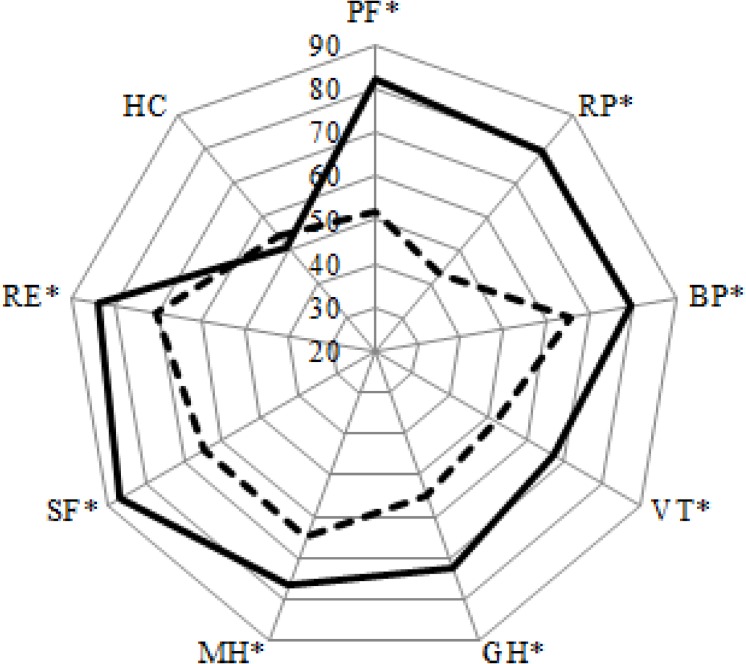
Health related quality of life in rehabilitation and healthy individuals [40]. Dotted line = rehabilitation patients; solid line = healthy reference population [40]. Asterisks indicate significant differences between the rehabilitation patients and healthy individuals. PF = physical functioning; SF = social functioning; RP = role limitations–physical; RE = role limitations–emotional; MH = mental health; VT = vitality; BP = pain; GH = general health; HC = health change.

**Fig 2:**
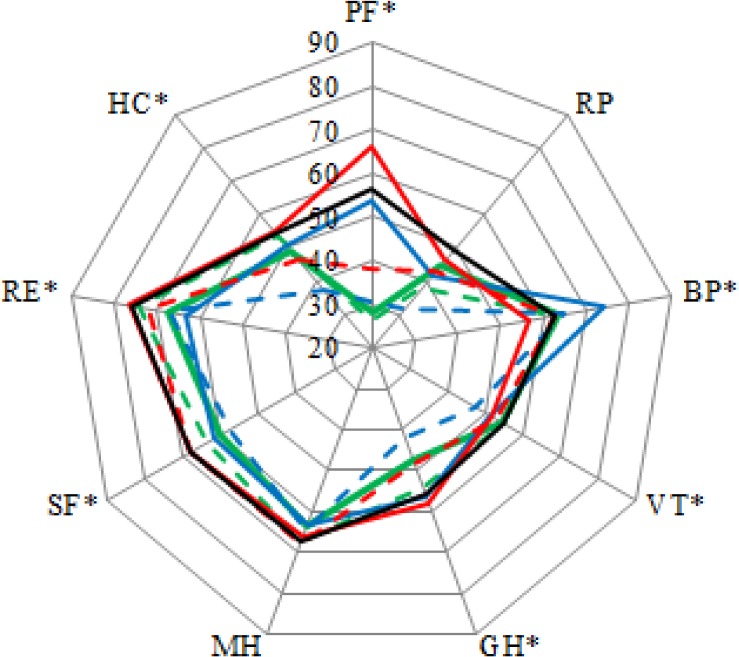
Health related quality of life separated for different diagnosis groups. Green solid line = amputation; green dotted line = spinal cord injury; blue solid line = brain injury; blue dotted line = multiple sclerosis; red solid line = chronic pain; red dotted line = other neurological disease; black line = other patients. Asterisks indicate significant differences between different diagnoses groups. PF = physical functioning; SF = social functioning; RP = role limitations–physical; RE = role limitations–emotional; MH = mental health; VT = vitality; BP = pain; GH = general health; HC = health change.

A total of 1223 patients (39%) completed the questionnaire (Table 1), of whom 1113 persons (91%) responded using the paper questionnaire, and 110 persons (9%) filled in the online questionnaire. Participants were older compared to non-participants (t = -8.903 (2746.7); p < 0.001). The distribution of diagnoses differed between participants and non-participants (X^2^ = 31.156 (6); p <0.001). No differences between participants and non-participants were found regarding gender (X^2^ = 0.821 (1); p = 0.365) and follow up period (t = 1.001 (3167); p = 0.317) (Table 1). Missing items per question ranged from 0.4 to 10.8%, and scores on subscales missed in 0.1 to 8.2% of the participants. HRQoL was significantly lower in the current rehabilitation population compared to the healthy reference population [40] on all subscales except for the health change subscale (Table 2, Fig 1). Results of the multiple regression analyses investigating the effect of follow up, gender, age, diagnosis and activity intensity on HRQoL are presented in Table 3. Follow-up negatively affected health change (p = 0.035), whereas age has a negative effect on physical functioning (p <0.001), social functioning (p = 0.004) and health change (p = 0.001). Diagnosis was affecting all subscales except for role limitations physical and mental health (p ranged <0.001 to 0.643). Movement intensity positively influenced all subscales except mental health (p ranged <0.001 to 0.190). The effect of diagnosis on the different subscales of the RAND-36 is displayed in Fig 2. Scores on the RAND-36 for the different subgroups of our rehabilitation population are presented in S1 Appendix.

**Table 1:** Characteristics of the participants and non-participants.

	Participants (n = 1223)	Non-participants (n = 1946)	Difference (95% CI)	p-value
	Mean±SD	Mean±SD		
Age (years) *	53.9±14.3	49.1±15.6	-4.8 (-5.9; -3.8)	<0.001
Follow up (months)	29.1±10.5	29.5±10.5	0.4 (-0.4; 1.1)	0.317
	n (%)	n (%)		
Gender (men)	609 (50)	931 (48)		0.365
Diagnosis *				<0.001
Amputation	49 (4)	77 (4)		
Brain injury ^a^	418 (34)	564 (29)		
Chronic pain	334 (27)	664 (34)		
Multiple sclerosis	73 (6)	98 (5)		
Spinal cord injury	98 (8)	67 (3)		
Other neurological disabilities ^b^	99 (8)	214 (11)		
Other disabilities ^c^	152 (12)	270 (14)		

* significant difference between participants and non-participants

^a^ Brain injuries from vascular, traumatic or oncological origin and meningitis

^b^ Spina bifida, Parkinson’s Disease and Guillain-Barré Syndrome

^c^ Disabilities such as tumors, fibromyalgia, arthritis, multi trauma, chronic fatigue syndrome and decubitus ulcer.

**Table 2:** Difference in HRQoL between the current rehabilitation population and a healthy Dutch reference population [40].

	Rehabilitation ^a^	Healthy ^a^^,^^b^	Difference	95% CI	t-value	p-value
PF	51.6±31.7	81.9±23.2	-30.3	28.03; 32.57	25.68	<0.001
SF	64.6±27.1	86.9±20.5	-22.3	20.34; 24.26	21.92	<0.001
RP	43.1±40.6	79.4±35.5	-36.3	33.11; 39.49	22.20	<0.001
RE	70.5±40.8	84.1±32.3	-13.6	10.53; 16.67	8.62	<0.001
MH	65.2±15.1	76.8±18.4	-11.6	10.21; 12.99	16.52	<0.001
VT	52.1±16.6	67.4±19.9	-15.3	13.78; 16.82	19.99	<0.001
BP	65.2±26.6	79.5±25.6	-14.3	12.16; 16.44	13.03	<0.001
GH	54.7±21.1	72.7±22.7	-18.0	16.19; 19.81	19.61	<0.001
HC	51.2±24.3	52.4±19.4	-1.2	-0.60; 3.00	1.29	0.197

^a^ mean±SD

^b^ Results of a Dutch reference population [40]; PF = physical functioning; SF = social functioning; RP = role limitations–physical; RE = role limitations–emotional; MH = mental health; VT = vitality; BP = pain; GH = general health; HC = health change; 95% CI = 95% confidence interval

**Table 3:** Results of the multiple regression analyses for all nine subscales of the RAND-36.

Subscale	Predictor	Coëfficiënt (SE)	95% CI	p-value	R^2^ change
PF	**Characteristics**			<0.001	0.075
	Follow-up ^a^	-0.8*10^−2^ (0.8*10^−1^)	-0.2; 0.2	0.922	
	Gender ^b^	1.5 (1.7)	-1.9; 4.9	0.401	
	Age ^c^	-0.5 (0.1)	-0.7; -0.4	<0.001	
	**Diagnosis** ^**d**^			<0.001	0.139
	Amputation	0.9 (6.3)	-11.4; 13.1	0.888	
	Spinal cord injury	-9.1 (5.3)	-19.5; 1.3	0.087	
	Brain injury ^e^	18.5 (4.1)	10.4; 26.6	<0.001	
	Chronic pain	29.9 (4.3)	21.5; 38.2	<0.001	
	Other neurological disability ^f^	4.8 (5.2)	-5.3; 14.9	0.354	
	Other ^g^	16.9 (4.7)	7.6; 26.1	<0.001	
	**Movement intensity**			<0.001	0.064
	Movement intensity ^h^	0.2 (0.3)	-0.3; 0.7	0.439	
	**Interaction terms**			<0.001	0.020
	Age * movement	0.7*10^−2^ (0.3*10^−2^)	0.2*10^−2^; 0.1*10^−1^	0.007	
	Amputation * movement	-0.6 (0.3)	-1.1; -0.7*10^−1^	0.028	
	Spinal cord injury * movement	-0.1 (0.3)	-0.6; 0.4	0.658	
	Brain injury * movement	-0.2*10^−1^ (0.2)	-0.5; 0.5	0.942	
	Chronic pain * movement	-0.3 (0.2)	-0.8; 0.2	0.200	
	Other neurological disability * movement	-0.2 (0.3)	-0.8; 0.3	0.848	
	Other * movement				
	**Constant**	55.8 (5.8)	44.5; 67.1	<0.001	
SF	**Characteristics**			0.019	0.010
	Follow-up	0.7*10^−1^ (0.8*10^−1^)	-0.1; 0.2	0.423	
	Gender	-1.3 (1.7)	-4.6; 2.1	0.467	
	Age	-0.2 (0.7*10^−1^)	-0.4; -0.7*10^−1^	0.004	
	**Diagnosis**			0.005	0.013
	Amputation	7.0 (6.2)	-5.1; 19.1	0.257	
	Spinal cord injury	4.8 (5.2)	-5.4; 15.0	0.357	
	Brain injury	-0.3 (4.1)	-8.4; 7.8	0.946	
	Chronic pain	8.3 (4.3)	-0.6*10^−1^; 16.7	0.052	
	Other neurological disability	9.2 (5.1)	-0.8; 19.3	0.072	
	Other	8.8 (4.7)	-0.4; 18.1	0.060	
	**Movement intensity**			<0.001	0.009
	Movement intensity	0.5*10^−1^ (0.3)	-0.5; 0.6	0.846	
	**Interaction terms**			<0.001	0.014
	Age * movement	0.6*10^−2^ (0.3*10^−2^)	0.1*10^−2^; 0.1*10^−1^	0.022	
	Amputation * movement	-0.5 (0.3)	-1.0; 0.8*10^−1^	0.094	
	Spinal cord injury * movement	-0.2 (0.3)	-0.7; 0.3	0.402	
	Brain injury * movement	-0.9*10^−1^ (0.2)	-0.6; 0.4	0.699	
	Chronic pain * movement	-0.3 (0.2)	-0.8; 0.2	0.225	
	Other neurological disability * movement	-0.2 (0.3)	-0.7; 0.3	0.486	
	Other * movement	-0.3 (0.2)	-0.8; 0.2	0.243	
	**Constant**	67.2 (5.7)	56.0; 78.5	<0.001	
RP	**Characteristics**			0.087	0.007
	Follow-up	0.1 (0.1)	-0.1; 0.4	0.375	
	Gender	-2.2 (2.7)	-7.5; 3.1	0.410	
	Age	-0.1 (0.1)	-0.3; 0.6*10^−1^	0.165	
	**Diagnosis**			0.343	0.004
	Amputation	5.3 (8.9)	-12.3; 22.9	0.554	
	Spinal cord injury	6.4 (7.5)	-8.4; 21.2	0.395	
	Brain injury	4.8 (5.8)	-6.5; 16.1	0.401	
	Chronic pain	4.2 (5.9)	-7.3; 15.7	0.472	
	Other neurological disability	0.5 (6.6)	-12.5; 13.4	0.946	
	Other	7.4 (6.4)	-5.7; 20.4	0.268	
	**Movement intensity**			0.001	0.021
	MET * hr/wk	0.3 (0.1)	0.2; 0.4	<0.001	
	**Constant**	32.3 (8.3)	22.0; 54.5	<0.001	
RE	**Characteristics**			0.088	0.007
	Follow-up	0.2 (0.1)	-0.3*10^−1^; 0.5	0.084	
	Gender	0.9; 2.7	-4.5; 6.3	0.744	
	Age	-0.1 (0.1)	-0.3; 0.1	0.297	
	**Diagnosis**			0.002	0.021
	Amputation	-0.8 (10.4)	-21.2; 19.6	0.938	
	Spinal cord injury	5.4 (8.5)	-11.4; 22.1	0.528	
	Brain injury	-10.2 (6.7)	-23.4; 2.9	0.127	
	Chronic pain	5.5 (6.8)	-8.0; 19.0	0.422	
	Other neurological disability	2.4 (8.2)	-13.7; 18.4	0.774	
	Other	3.9 (7.7)	-11.2; 18.9	0.615	
	**Movement intensity**			<0.001	0.006
	Movement intensity	0.2*10^−1^ (0.4)	-0.7; 0.7	0.957	
	**Interaction terms**			0.004	0.003
	Amputation * movement	0.3 (0.5)	-0.6; 1.2	0.476	
	Spinal cord injury * movement	0.2*10^−1^ (0.4)	-0.8; 0.8	0.954	
	Brain injury * movement	0.2 (0.4)	-0.5; 1.0	0.546	
	Chronic pain * movement	0.3*10^−1^ (0.4)	-0.7; 0.8	0.945	
	Other neurological disability * movement	0.9*10^−1^ (0.4)	-0.7; 0.9	0.824	
	Other * movement	0.2 (0.4)	-0.6; 1.0	0.595	
	**Constant**	66.7 (8.8)	49.4; 84.0	<0.001	
MH	**Characteristics**			0.598	0.002
	Follow-up	0.6*10^−1^ (0.5*10^−1^)	-0.3*10^−1^; 0.2	0.189	
	Gender	-0.7 (1.0)	-2.6; 1.3	0.502	
	Age	0.2*10^−1^ (0.4*10^−1^)	-0.5*10^−1^; 0.9*10^−1^	0.601	
	**Diagnosis**			0.643	0.005
	Amputation	1.5 (3.2)	-4.7; 7.7	0.642	
	Spinal cord injury	1.2 (2.7)	-4.1; 6.4	0.668	
	Brain injury	-1.0 (2.1)	-5.1; 3.1	0.632	
	Chronic pain	1.1 (2.1)	-3.1; 5.3	0.617	
	Other neurological disability	1.5 (2.4)	-3.3; 6.2	0.545	
	Other	1.3 (2.4)	-3.4; 6.1	0.578	
	**Movement intensity**			0.190	0.007
	Movement intensity	0.6*10^−1^ (0.2*10^−1^)	0.2*10^−1^; 0.1	0.010	
	**Constant**	61.1 (3.0)	55.3; 67.0	<0.001	
VT	**Characteristics**			0.031	0.009
	Follow-up	0.9*10^−1^ (0.5*10^−1^)	-0.9*10^−2^; 0.2	0.076	
	Gender	1.1 (1.1)	-0.9; 3.2	0.278	
	Age	-0.2*10^−1^ (0.4*10^−1^)	-0.9*10^−1^; 0.5*10^−1^	0.623	
	**Diagnosis**			0.004	0.015
	Amputation	9.5 (3.7)	2.1; 16.9	0.011	
	Spinal cord injury	6.8 (3.2)	0.6; 13.1	0.031	
	Brain injury	2.4 (2.5)	-2.5; 7.3	0.340	
	Chronic pain	2.7 (2.6)	-2.4; 7.8	0.299	
	Other neurological disability	4.3 (3.4)	-1.9; 10.4	0.176	
	Other	4.1 (2.9)	-1.5; 9.8	0.151	
	**Movement intensity**			<0.001	0.033
	Movement intensity	0.3 (0.1)	0.3*10^−1^; 0.6	0.030	
	**Interaction terms**			<0.001	0.005
	Amputation * movement	-0.2 (0.2)	-0.5; 0.2	0.317	
	Spinal cord injury * movement	-0.2 (0.2)	-0.5; 0.9*10^−1^	0.167	
	Brain injury * movement	-0.1 (0.1)	-0.4; 0.1	0.321	
	Chronic pain * movement	-0.2 (0.1)	-0.5; 0.8*10^−1^	0.147	
	Other neurological disability * movement	-0.8*10^−1^ (0.2)	-0.4; 0.2	0.603	
	Other * movement	-0.9*10^−1^ (0.2)	-0.4; 0.2	0.554	
	**Constant**	44.0 (3.3)	37.6; 50.4	<0.001	
BP	**Characteristics**			0.016	0.010
	Follow-up	0.4*10^−1^ (0.8*10^−1^)	-0.1; 0.2	0.598	
	Gender	3.0 (1.7)	-0.3; 6.3	0.073	
	Age	-0.7*10^−1^ (0.6*10^−1^)	-0.2; 0.4*10^−1^	0.218	
	**Diagnosis**			<0.001	0.063
	Amputation	-0.7 (5.9)	-12.3; 11.0	0.910	
	Spinal cord injury	-3.4 (5.0)	-13.2; 6.4	0.494	
	Brain injury	6.1 (4.0)	-1.7; 13.8	0.128	
	Chronic pain	-11.1 (4.1)	-19.2; -3.1	0.007	
	Other neurological disability	-3.9 (4.9)	-13.6; 5.7	0.426	
	Other	-7.2 (4.5)	-16.1; 1.7	0.114	
	**Movement intensity**			<0.001	0.013
	Movement intensity	0.3 (0.2)	-0.2; 0.7	0.215	
	**Interaction terms**			<0.001	0.005
	Amputation * movement	-0.3 (0.3)	-0.9; 0.2	0.200	
	Spinal cord injury * movement	-0.3 (0.3)	-0.8; 0.2	0.293	
	Brain injury * movement	-0.8*10^−1^ (0.2)	-0.5; 0.4	0.732	
	Chronic pain * movement	-0.1 (0.2)	-0.6; 0.3	0.648	
	Other neurological disability * movement	-0.2 (0.3)	-0.7; 0.3	0.415	
	Other * movement	0.4*10^−1^ (0.2)	-0.5; 0.4	0.874	
	**Constant**	65.7 (5.2)	55.5; 75.9	<0.001	
GH	**Characteristics**			0.015	0.010
	Follow-up	0.7*10^−1^ (0.6*10^−1^)	-0.5*10^−1^; 0.2	0.231	
	Gender	-2.3 (1.3)	-4.9; 0.2	0.076	
	Age	-0.7*10^−1^ (0.5*10^−1^)	-0.2; 0.2*10^−1^	0.113	
	**Diagnosis**			<0.001	0.035
	Amputation	8.2 (4.7)	-0.9; 17.4	0.078	
	Spinal cord injury	13.7 (3.9)	6.0; 21.4	0.001	
	Brain injury	11.3 (3.1)	5.2; 17.4	<0.001	
	Chronic pain	13.4 (3.2)	7.0; 19.7	<0.001	
	Other neurological disability	7.0 (3.9)	-0.5; 14.6	0.069	
	Other	8.9 (3.6)	1.9; 15.8	0.013	
	**Movement intensity**			<0.001	0.039
	Movement intensity	0.2 (0.2)	-0.2; 0.5	0.278	
	**Interaction terms**			<0.001	0.007
	Amputation * movement	0.3*10^−1^ (0.2)	-0.4; 0.4	0.894	
	Spinal cord injury * movement	-0.1 (0.2)	-0.5; 0.3	0.609	
	Brain injury * movement	0.9*10^−1^ (0.2)	-0.3; 0.5	0.611	
	Chronic pain * movement	-0.4*10^−1^ (0.2)	-0.4; 0.3	0.835	
	Other neurological disability * movement	-0.1 (0.2)	-0.5; 0.3	0.549	
	Other * movement	0.1 (0.2)	-0.2; 0.5	0.489	
	**Constant**	43.4 (4.1)	35.4; 51.5	<0.001	
HC	**Characteristics**			<0.001	0.026
	Follow-up	-0.2 (0.9*10^−1^)	-0.4; -0.1*10^−1^	0.035	
	Gender	-0.8 (1.5)	-3.8; 2.1	0.581	
	Age	-0.2 (0.6*10^−1^)	-0.3; -0.8*10^−1^	0.001	
	**Diagnosis**			<0.001	0.025
	Amputation	16.9 (5.3)	6.4; 27.4	0.002	
	Spinal cord injury	16.2 (4.5)	7.3; 25.1	<0.001	
	Brain injury	14.2 (3.6)	7.2; 21.3	<0.001	
	Chronic pain	16.7 (3.7)	9.4; 23.9	<0.001	
	Other neurological disability	10.9 (4.5)	2.2; 19.7	0.014	
	Other	18.5 (4.1)	10.5; 26.5	<0.001	
	**Movement intensity**			<0.001	0.042
	Movement intensity	0.7 (0.3)	0.2; 1.3	0.004	
	**Interaction terms**			<0.001	0.014
	Follow up * movement	-0.6*10^−2^ (0.3*10^−2^)	-0.1*10^−1^; 0.1*10^−2^	0.075	
	Age * movement	0.4*10^−2^ (0.2*10^−2^)	<0.1*10^−2^; 0.9*10^−2^	0.078	
	Amputation * movement	-0.6 (0.5)	-1.1; -0.2	0.009	
	Spinal cord injury * movement	-0.5 (0.2)	-1.0; -0.6*10^−1^	0.026	
	Brain injury * movement	-0.5 (0.2)	-0.9; -0.9*10^−1^	0.016	
	Chronic pain * movement	-0.5 (0.2)	-0.9; -0.1	0.010	
	Other neurological disability * movement	-0.5 (0.2)	-0.9; -0.2*10^−1^	0.043	
	Other * movement	-0.6 (0.2)	-1.0; -0.2	0.007	
	**Constant**	48.9 (5.2)	38.7; 59.1	<0.001	

^a^ Follow-up in months

^b^ Reference = female

^c^ Age in years

^d^ Reference = Multiple sclerosis

^e^ Brain injuries from vascular, traumatic or oncological origin and meningitis

^f^ Spina bifida, Parkinson’s Disease and Guillain-Barré Syndrome

^g^ Disabilities such as tumors, fibromyalgia, arthritis, multi trauma, chronic fatigue syndrome and decubitus ulcer

^h^ Movement intensity expressed as MET * hr/wk; PF = physical functioning; SF = social functioning; RP = role limitations–physical; RE = role limitations–emotional; MH = mental health; VT = vitality; BP = pain; GH = general health; HC = health change; SE = standard error; 95% CI = 95% confidence interval.

## Discussion

This study aimed to establish the HRQoL as measured using the RAND-36 in a Dutch rehabilitation population, and to identify factors influencing HRQoL in this diverse population. Participation in this study was 39%. This percentage is lower than in a study in both Dutch community dwelling and chronic disease populations of which a part was face-to-face interviewed [44], and little higher compared to a study in healthy individuals in the Netherlands [45]. Participants in the current study were ex-patients, and thereby familiar with our institute. Presumably, this may explain the little higher response rate in the current study compared to the earlier study in the Netherlands [45]. Non-responding may lead to non-response bias. From baseline characteristics it is known that participants differed from non-participants only on age and diagnosis.

Participants were significantly older compared to non-participants. This is in agreement with overall trends in questionnaire based research [46], and is suggested to be explained by age-related moral differences on responding questionnaires. From the multiple regression analyses it can be concluded that age negatively affects scores on physical functioning, social functioning, general health and health change. Therefore, it is plausible that results on these subscales are underestimated by the representation of the sample. However, the highest coefficient on age was -0.5, and since the age differed only 5.1 years on average between the participants and non-participants, this will maximally have an effect of -2.6 on a scale ranging 0 to 100.

The group of participants included substantially more brain injury and SCI patients, and less patients suffering from chronic pain and other neurological diseases compared to the non-participants. However, by the large number of participants and the relatively small differences in coefficients in the multiple regression analysis between these specific diagnoses, we assume that the different distribution of diagnoses between the participants and non-participants was not substantially influencing the results of this study.

Our rehabilitation population scored lower on all subscales except for the health change subscale, when comparing to a healthy Dutch reference population (Table 2, Fig 1) [40]. This seems straightforward, because of the physical disabilities that the rehabilitation population suffer with, and is in accordance with earlier research [9,10,13–15,25,34,38]. In chronic disease patients, the discrimination for clinically relevant changes in HRQoL is indicated to be 0.5 SD [47]. Using this indication, the significant differences mentioned above are also clinically relevant. Compared to the rehabilitation population, the reference population was younger and consisted of a higher proportion of females. However, we think these differences will not have a large impact on comparisons made, since the coefficients of age and gender found in the multiple regression analyses are very small relatively compared to the differences between the populations. No significant differences were found on the health change subscale between the two populations. This implies that the health of the rehabilitation patients did not change faster or slower than that of the healthy population. This seems evident since the included rehabilitation patients are in the chronic phase, in which no major changes are expected anymore. When comparing the current results with the reference values, a time difference of approximately 20 years between these two measures, with probably an associated change on health perspective has to be taken into account. However, differences between both groups are that large that we assume this time difference will not have biased the conclusions.

Multiple regression analyses (Table 3) showed that follow-up only influenced scores on health change. Health change was negatively affected by follow-up period, which implies that a longer follow-up leads to a smaller health change. This finding is clinical probable since the amount of progression stagnates over time. Gender was not associated with any of the subscales, which is in accordance with findings in healthy people [40]. Age had a negative effect on physical functioning, social functioning and health change, what indicates that level of physical functioning and social functioning are lower at higher age, and that health changes on a slower pace at higher age. The effects on physical functioning and health change are in accordance with findings in healthy people [40]. In healthy people age did not affected social functioning [40].

Diagnosis affected outcomes on all subscales except for role limitations physical and mental health. However, due to the large sample size not all statistically significant differences are clinically relevant. Following the rule of the thumb of half a SD [47], clinically relevant differences between diagnoses were found only on physical functioning, pain, general health and health change (S1 Appendix, Fig 2). It is remarkable that all these subscales are predominantly in the physical field. So unless rehabilitation patients in general have decreased psychosocial status compared to healthy individuals, no clinically relevant differences between the diagnoses can be found on those psychosocial subscales. In general, MS patients have a relatively low HRQoL and chronic pain patients have a relatively high HRQoL (Fig 2). This is contrary to findings in rehabilitation outpatients, in which chronic pain patients scored lower compared to other diagnoses [8]. Chronic pain patients in the current study form a too positive representation of the chronic pain population, since only chronic pain patients in which progress is expected, that with higher physical functioning in general, are admitted for rehabilitation and thus were included in the current study.

Movement intensity was positively related to outcomes on all subscales except for mental health. This implies that being more physically active is associated with an increased HRQoL in bio-, psycho- and social domains. The positive effect of movement intensity can be due to the energy cost of the activity performed (amount of MET) or by the duration of the activity (hours/week). However, since no causal relationship can be established through the cross-sectional design of this study, it can also be that people with a higher HRQoL are more physically active. Coefficients of movement intensity appear to be relatively small, however the mean metabolic equivalent (MET value) of the activities performed by the active part of the group was 4.9. Performing one hour of such an average activity per week, would thereby increase the score on the subscale with 4.9 multiplied by the coefficient. In this study the 2011 compendium of Ainsworth was used to connect MET values to specific activities [43]. However, values in this compendium are calculated for healthy people. No MET values appropriate for people with physical disabilities were available.

A significant positive interaction effect between age and movement intensity was found on physical functioning and social functioning. This implies that physical activity has a stronger effect on physical- and social functioning in older people compared to younger people. Moreover, significant interaction effects were found between several diagnoses and movement intensity on physical functioning and health change. All significant interaction effects were negative, which implies that movement is affecting physical functioning and health change less, in people suffering from that diagnosis compared to MS patients (reference population). To the best of our knowledge this is the first study investigating these interactions, whereby it was not possible to compare the current results to earlier findings.

The explained variances of the multiple regression analyses range from 1.3% in the mental health subscale to 29.8% in the physical functioning subscale. Especially the explained variance by diagnoses is not very high in some subscales, which may be related to variability within diagnoses groups (severity of injury, level of amputation or level of SCI). Due to the large number of different diagnoses in this study, we have chosen to distinguish diagnosis only. Subdividing based on severity of injury would have led to even more (small) subgroups.

The aim of this study was to overview the HRQoL of the Dutch rehabilitation population. By the diverse diagnoses in this study, results of this study provide insights in differences on HRQoL between those different diagnoses. This study is limited by the fact that all included patients were treated in one rehabilitation center in the north of the Netherlands, and almost all participants live in the north of the country. The results of this study may not be generalizable to the entire Dutch rehabilitation population. As stated above, by the diverse population measured, it was not possible to distinguish severity within diagnoses. As an extension of the current study, future research could focus on specific diagnoses and could more in depth (regarding severity) investigate predictors of HRQoL. Hereby more precise indication values can be formed, which can be useful in setting treatment goals. Additionally prospective research is needed to study the causality of the relationship between movement intensity and HRQoL.

## Conclusions

HRQoL in rehabilitation patients was lower compared to that of healthy people. Diagnosis predominantly affects physical functioning, pain, general health and health change. There is a strong relationship between movement intensity and almost all fields of HRQoL. Moreover, in a clinical rehabilitation setting the results of this study can be used as reference values for HRQoL.

## Supporting Information

S1 Appendix Health Related Quality of Life in a Dutch rehabilitation population separated for different subgroups. PF = physical functioning; SF = social functioning; RP = role limitations–physical; RE = role limitations–emotional; MH = mental health; VT = vitality; BP = bodily pain; GH = general health; HC = health change; y = years; Amp. = amputation; SCI = spinal cord injury; MS = multiple sclerosis; neuro other = other neurological diseases; * clinical relevant difference between groups (difference > 0.5 SD [47]), pooled SDs can be found in Table 2; ^a^ Brain injuries from vascular, traumatic or oncological origin and meningitis; ^b^ Spina bifida, Parkinson’s Disease and Guillain-Barré Syndrome; ^c^ Disabilities such as tumors, fibromyalgia, arthritis, multi trauma, chronic fatigue syndrome and decubitus ulcer.
(PDF)

S1 Database (PDF)
