# Supplementary material for: Health Related Quality of Life in a Dutch Rehabilitation Population: Reference Values and the Effect of Physical Activity
Source: PLoS One. 2017 Jan 6;12(1):e0169169. doi: 10.1371/journal.pone.0169169 (PMC5217970; doi:10.1371/journal.pone.0169169)
Supplement: S1 Appendix — PF = physical functioning; SF = social functioning; RP = role limitations–physical; RE = role limitations–emotional; MH = mental health; VT = vitality; BP = bodily pain; GH = general health; HC = health change; y = years; Amp. = amputation; SCI = spinal cord injury; MS = multiple sclerosis; neuro other = other neurological diseases; * clinical relevant difference between groups (difference > 0.5 SD [47]), pooled SDs can be found in Table 2; a Brain injuries from vascular, traumatic or oncological origin and meningitis; b Spina bifida, Parkinson’s Disease and Guillain-Barré Syndrome; c Disabilities such as tumors, fibromyalgia, arthritis, multi trauma, chronic fatigue syndrome and decubitus ulcer. (PDF) [file pone.0169169.s001.pdf]

S1 Appendix. Health Related Quality of Life in a Dutch rehabilitation population separated for different subgroups

	Male	Female
PF	52.8±32.1	54.7±30.0
SF	65.6±27.0	65.1±26.6
RP	44.0±41.0	43.1±40.4
RE	70.3±40.8	70.9±40.6
MH	65.3±15.5	65.8±14.4
VT	53.8±17.1	50.9±15.9
BP	68.5±26.3	62.8±26.1
GH	54.9±21.7	55.8±20.9
HC	52.3±24.0	52.1±24.5

	18-24 y	25-35 y	35-44 y	45-54 y	55-64 y	65-74 y	75+ y
PF*	59.0±29.5	65.3±28.5	59.3±30.4	57.9±29.0	50.0±30.8	47.0±33.0	30.8±29.1
SF	74.4±28.2	71.9±23.3	64.2±28.8	64.7±27.3	62.9±25.5	66.5±26.2	61.9±29.6
RP	51.3±36.3	52.5±40.6	44.8±39.9	43.5±41.7	40.0±38.9	44.9±42.5	37.5±39.5
RE	76.1±39.7	74.5±37.6	74.9±38.9	68.6±41.5	70.5±40.3	69.1±41.7	61.9±46.9
MH	65.4±17.9	66.7±13.9	65.6±13.7	64.2±15.3	64.8±15.2	67.4±14.8	65.3±16.1
VT	56.3±17.2	55.2±14.0	51.0±15.7	50.7±16.7	51.3±16.5	54.5±17.8	51.6±17.1
BP	65.0±27.2	69.4±23.8	63.0±28.4	64.1±26.2	66.8±25.5	66.4±26.0	69.7±29.0
GH	62.8±20.8	60.6±23.0	53.9±21.1	56.1±21.9	53.3±20.9	53.6±21.5	55.7±17.7
HC	53.9±16.8	56.2±25.6	50.9±23.6	53.3±23.8	51.4±24.0	51.3±26.3	44.1±22.0

	Amp.	SCI	Brain injury [a]	MS	Chronic pain	Neuro other [b]	Other [c]
PF*	27.9±21.4	26.1±27.9	53.8±32.0	30.6±27.5	66.1±23.6	37.9±31.1	56.5±31.9
SF	60.0±32.1	63.9±26.3	61.6±27.1	57.7±24.6	68.2±25.6	68.1±25.8	67.7±29.6
RP	44.6±41.9	38.0±40.8	41.4±39.6	31.8±38.4	46.3±41.3	42.8±42.1	49.1±40.7
RE	67.5±40.3	74.0±39.4	63.4±43.1	67.2±41.6	76.2±38.3	72.1±40.8	76.0±37.9
MH	63.4±20.0	67.1±13.8	63.0±15.3	64.6±13.8	65.9±14.6	67.9±13.3	67.5±15.7
VT	54.7±20.8	55.0±15.7	51.3±16.9	47.2±15.2	51.4±15.9	53.2±15.8	54.9±17.0
BP*	63.2±29.3	62.9±25.0	74.1±25.8	65.0±28.2	56.9±23.1	62.5±26.6	62.8±28.6
GH*	47.7±24.5	54.2±20.1	56.2±20.8	42.2±19.9	58.2±20.5	48.1±19.1	56.1±21.0
HC*	49.0±23.9	53.4±25.2	50.5±22.2	37.0±24.0	54.8±24.7	46.4±26.4	54.3±24.5

PF = physical functioning; SF = social functioning; RP = role limitations – physical; RE = role limitations – emotional; MH = mental health; VT = vitality; BP = bodily pain; GH = general health; HC = health change; y = years; Amp. = amputation; SCI = spinal cord injury; MS = multiple sclerosis; neuro other = other neurological diseases; * clinical relevant difference between groups (difference > 0.5 SD [47]), pooled SDs can be found in Table 2; [a] Brain injuries from vascular, traumatic or oncological origin and meningitis; [b] Spina bifida, Parkinson's Disease and Guillain-Barré Syndrome; [c] Disabilities such as tumours, fibromyalgia, arthritis, multi trauma, chronic fatigue syndrome and decubitus ulcer.
