# Supplementary material for: Health Related Quality of Life in a Dutch Rehabilitation Population: Reference Values and the Effect of Physical Activity
Source: PLoS One. 2017 Jan 6;12(1):e0169169. doi: 10.1371/journal.pone.0169169 (PMC5217970; doi:10.1371/journal.pone.0169169)
Supplement: S1 Database — (PDF) [file pone.0169169.s002.pdf]

**S1 Database.**

| Diagnosis | Movement intensity | PF | SF | RP | RE | MH | VT | BP | GH | HC |
|---|---|---|---|---|---|---|---|---|---|---|
| 1 | 0 | 45 | 100 | 75 | 100 | 72 | 80 | 100 | 55 | 50 |
| 3 | 0 | 70 | 50 | 0 | | 52 | 35 | | 40 | 50 |
| 3 | 0 | 0 | 38 | 0 | 100 | 64 | 45 | 45 | 55 | 0 |
| 3 | 100.5 | 85 | 63 | 50 | 100 | 76 | 65 | 100 | 50 | 75 |
| 3 | 0 | 95 | 100 | | | 44 | 55 | 90 | 50 | 100 |
| 3 | 32.25 | 90 | 38 | 25 | 67 | 48 | 25 | 100 | 55 | 50 |
| 5 | 0 | 70 | 13 | 0 | 100 | 32 | 30 | 0 | 30 | 50 |
| 7 | 7 | 20 | 0 | 50 | 0 | 48 | 45 | 22 | 50 | 50 |
| 3 | 0 | 70 | 50 | 25 | 0 | 56 | 50 | 100 | 45 | 50 |
| 5 | 0 | 80 | 88 | 100 | 100 | 80 | 70 | 100 | 80 | 50 |
| 3 | 0 | 75 | 38 | 0 | 33 | 44 | 35 | 100 | 50 | 50 |
| 6 | 0 | 5 | 25 | 0 | 0 | 56 | 50 | 22 | 35 | 25 |
| 5 | 3.13 | 100 | 100 | 75 | 100 | 72 | 55 | 57 | 80 | 25 |
| 5 | 11.5 | 55 | 63 | 0 | 33 | 72 | 40 | 45 | 50 | 50 |
| 2 | 0 | 15 | 38 | 0 | 0 | 56 | 55 | 22 | 75 | 50 |
| 5 | 0 | 15 | 50 | 0 | 0 | 32 | 25 | 22 | 30 | 25 |
| 3 | 34.5 | 85 | 75 | 0 | 0 | 48 | 50 | 67 | 60 | 75 |
| 7 | 9 | 15 | 25 | 0 | 100 | 76 | 15 | 100 | 50 | 50 |
| 5 | 12 | 60 | 100 | 75 | 100 | 84 | 90 | 57 | 80 | 50 |
| 4 | 21.4 | 30 | 100 | 100 | 100 | 84 | 65 | 90 | 35 | 100 |
| 3 | 31.55 | 90 | 63 | 0 | 100 | 72 | 50 | 100 | 45 | 50 |
| 3 | 0 | 95 | 75 | 0 | 100 | 60 | 45 | 100 | 45 | 25 |
| 5 | 28.5 | 95 | 88 | 100 | 100 | 80 | 80 | 90 | 85 | 75 |
| 3 | 10.25 | 75 | 63 | 0 | 0 | 64 | 40 | 78 | 35 | 50 |
| 7 | 12.75 | 20 | 38 | 25 | 0 | 56 | 60 | 22 | 50 | 50 |
| 4 | 0 | 15 | 25 | 25 | 100 | 56 | 50 | 35 | 25 | 25 |
| 2 | 0 | 95 | 75 | 25 | 33 | 64 | 50 | 67 | 50 | 50 |
| 3 | 75 | 100 | 88 | 100 | 100 | 64 | 65 | 100 | 75 | 50 |
| 3 | 0 | 75 | 63 | 0 | 0 | 60 | 60 | 67 | 55 | 75 |
| 1 | 0 | 20 | 63 | | | 52 | 40 | 67 | 25 | 25 |
| 4 | 0 | 20 | 88 | 50 | 67 | 64 | 40 | 47 | 35 | 25 |
| 3 | 47.5 | 95 | 88 | 50 | 33 | 80 | 80 | 100 | 90 | 50 |
| 5 | 22.5 | 70 | 63 | 0 | 67 | 56 | 35 | 43 | 35 | 25 |
| 2 | 0 | | 75 | | | 48 | 45 | 57 | 35 | 75 |
| 3 | 0 | 100 | 100 | 100 | 100 | 76 | 75 | 100 | 85 | 75 |
| 3 | 19 | 45 | 63 | 75 | 100 | 72 | 60 | 100 | 70 | 75 |
| 2 | 0 | | 100 | | | 84 | 80 | 100 | 70 | 50 |
| 7 | 30.45 | 95 | 100 | 75 | 100 | 80 | 65 | 90 | 75 | 100 |
| 3 | 34.4 | 60 | 38 | 0 | 67 | 60 | 45 | 67 | 50 | 50 |
| 4 | 12.32 | 95 | 63 | 0 | 100 | 76 | 60 | 100 | 65 | 50 |
| 3 | 0 | 85 | 75 | 50 | | 64 | 50 | 100 | 45 | 50 |
| 3 | 18.8 | 20 | 100 | 100 | 100 | 68 | 75 | 100 | 95 | 50 |
| 3 | 15.25 | 40 | 75 | 100 | 100 | 64 | 45 | 90 | 60 | 50 |
| 3 | 13 | 45 | 25 | 0 | 100 | 80 | 50 | 100 | 50 | 50 |
| 5 | 5 | 65 | 63 | 50 | 100 | 68 | 45 | 57 | 50 | 50 |
| 4 | 12 | 15 | 75 | 100 | 100 | 60 | 55 | 45 | 65 | 50 |
| 7 | 18.75 | 70 | 88 | 0 | 100 | 80 | 65 | 33 | 45 | 100 |
| 7 | 0 | 0 | 13 | 0 | 0 | 16 | 15 | 45 | 5 | 0 |
| 6 | 0 | 20 | 13 | 0 | 33 | 56 | 45 | 10 | 15 | 25 |
| 7 | 0 | 10 | 38 | | 67 | 56 | 30 | 33 | 25 | 0 |
| 3 | 7.5 | 15 | 50 | 25 | 33 | 56 | 50 | 55 | 50 | 50 |
| 5 | 39.5 | 55 | 25 | 0 | 0 | 44 | 35 | 22 | 25 | 75 |
| 3 | 35 | 65 | 88 | 75 | 100 | 80 | 65 | 67 | 70 | 50 |
| 4 | 0 | 20 | 75 | 0 | 100 | 64 | 60 | 80 | 60 | 50 |
| 5 | 24 | 60 | 88 | 0 | 100 | 80 | 50 | 57 | 50 | 100 |
| 5 | 16 | 50 | 100 | 50 | 100 | 84 | 60 | 67 | 50 | 50 |

| | | | | | | | | | | |
|---|---|---|---|---|---|---|---|---|---|---|
| 3 | 30.5 | 40 | 50 | 50 | 67 | 64 | 35 | 100 | 85 | 75 |
| 3 | 0 | 80 | 38 | 0 | 0 | 60 | 55 | 100 | 35 | 0 |
| 2 | 0 | 5 | 25 | 25 | 100 | 72 | 75 | 57 | 100 | 50 |
| 2 | 0 | | 75 | 50 | 100 | 64 | 65 | 57 | 75 | 75 |
| 7 | 47.2 | 100 | 75 | 100 | 100 | 64 | 55 | 80 | 75 | 50 |
| 3 | 0 | 0 | 63 | 25 | 100 | 64 | 50 | 57 | 80 | 25 |
| 3 | 0 | 5 | 13 | 0 | 0 | 68 | 65 | 100 | 50 | 25 |
| 3 | 6 | 35 | 88 | 0 | 100 | 76 | 50 | 67 | 55 | 50 |
| 5 | 18.25 | 70 | 88 | 100 | 67 | 76 | 70 | 80 | 75 | 50 |
| 5 | 0 | 40 | 75 | 0 | 100 | 68 | 65 | 55 | 45 | 25 |
| 3 | 8.7 | 40 | 88 | 100 | 67 | 76 | 75 | 100 | 75 | 50 |
| 5 | 35.5 | 70 | 63 | 75 | 100 | 80 | 60 | 45 | 55 | 100 |
| 3 | 7 | 15 | 38 | 0 | 0 | 44 | 25 | 45 | 25 | 50 |
| 6 | 0 | 45 | 63 | 100 | 0 | 64 | 55 | 80 | 55 | 75 |
| 5 | 9.8 | 65 | 75 | 0 | 33 | 56 | 55 | 45 | 75 | 25 |
| 3 | 0 | 0 | 38 | 75 | 0 | 52 | 50 | 33 | 55 | 25 |
| 3 | 17.9 | 75 | 75 | 0 | | 72 | 60 | 78 | 65 | 50 |
| 3 | 0 | 65 | 75 | 0 | 0 | 88 | 55 | 78 | 45 | 100 |
| 3 | 14 | 100 | 50 | 0 | 100 | 68 | 45 | 100 | 60 | 50 |
| 3 | 27.6 | 70 | 50 | 0 | 100 | 68 | 40 | 100 | 60 | 50 |
| 2 | 6.45 | | 88 | 50 | 100 | 84 | 85 | 100 | 80 | 50 |
| 7 | 0 | 0 | 0 | 0 | 100 | 80 | 10 | 0 | 0 | 0 |
| 5 | 21 | 100 | 100 | 100 | 100 | 76 | 85 | 100 | 95 | 100 |
| 4 | 12.3 | 35 | 63 | 0 | 100 | 60 | 35 | 22 | 30 | 25 |
| 6 | 0 | 20 | 63 | | 67 | 76 | 40 | 80 | 30 | 25 |
| 5 | 36 | 90 | 63 | 75 | 100 | 64 | 60 | 57 | 60 | 50 |
| 7 | 19.8 | 70 | 75 | 100 | 100 | 76 | 65 | 67 | 75 | 50 |
| 1 | 8.6 | 10 | 50 | 25 | 0 | 36 | 40 | 57 | 40 | 50 |
| 3 | 0 | 25 | 63 | 0 | 0 | 52 | 45 | 55 | 35 | 25 |
| 5 | 14.5 | 95 | 88 | 100 | 100 | 60 | 55 | 78 | 60 | 50 |
| 5 | 30 | 60 | 38 | 0 | 67 | 52 | 30 | 22 | 40 | 50 |
| 5 | 19.8 | 95 | 100 | 100 | 100 | 88 | 65 | 100 | 95 | 100 |
| 3 | 0 | 35 | 75 | 25 | 100 | 76 | 45 | 20 | 25 | 50 |
| 3 | 55.1 | 80 | 100 | 100 | 100 | 80 | 65 | 90 | 50 | 25 |
| 3 | 0 | 50 | 25 | 0 | 100 | 80 | 80 | 33 | 75 | 50 |
| 5 | 0 | 80 | 63 | 0 | 67 | 60 | 50 | 67 | 50 | 100 |
| 3 | 16.5 | 70 | 38 | 25 | 100 | 68 | 40 | 45 | 40 | 50 |
| 5 | 62 | 85 | 88 | 25 | 100 | 72 | 50 | 57 | 80 | 75 |
| 3 | 0 | 20 | 25 | 0 | 0 | 44 | 30 | 55 | 40 | 0 |
| 3 | 19.6 | 55 | 50 | 0 | 100 | 72 | 45 | 57 | 40 | 75 |
| 5 | 26 | 80 | 88 | 100 | 100 | 76 | 60 | 67 | 85 | 100 |
| 7 | 9 | 70 | 88 | 100 | 100 | 76 | 60 | 90 | 65 | 50 |
| 3 | 0 | 10 | 63 | 50 | 100 | 72 | 55 | 100 | 55 | 50 |
| 3 | 0 | 50 | 100 | | | 80 | 35 | 57 | 60 | 0 |
| 5 | 19 | 75 | 63 | 0 | 100 | 60 | 60 | 45 | 60 | 75 |
| 7 | 0 | 10 | 25 | | | 64 | 50 | 22 | 55 | 75 |
| 3 | 0 | 0 | 50 | 25 | 33 | 68 | 25 | 45 | 35 | 25 |
| 5 | 0 | 65 | 100 | 50 | 0 | 56 | 60 | 57 | 80 | 0 |
| 2 | 21.35 | 0 | 38 | 0 | 0 | 44 | 40 | 22 | 20 | 50 |
| 4 | 0 | 55 | 63 | 0 | 0 | 44 | 35 | 43 | 40 | 25 |
| 3 | 0 | 95 | 50 | 0 | 67 | 68 | 55 | 45 | 35 | 100 |
| 5 | 22.9 | 90 | 75 | 100 | 100 | 64 | 65 | 78 | 70 | 75 |
| 4 | 14 | 60 | 63 | 75 | 100 | 52 | 50 | 57 | 40 | 25 |
| 7 | 0 | 25 | 0 | 0 | 100 | 68 | 30 | 33 | 30 | 0 |
| 3 | 52.5 | 100 | 88 | 100 | 100 | 64 | 70 | 100 | 70 | 50 |
| 3 | 6 | 10 | 0 | 0 | 100 | 60 | 20 | 31 | 35 | 25 |
| 3 | 10.9 | 95 | 100 | 100 | 100 | 56 | 45 | 100 | 55 | 50 |
| 5 | 0 | 15 | 13 | 0 | 100 | 64 | 35 | 10 | 55 | 50 |
| 3 | 46.95 | 55 | 63 | 0 | 100 | 80 | 60 | 45 | 85 | 100 |

| | | | | | | | | | | |
|---|---|---|---|---|---|---|---|---|---|---|
| 5 | 14 | 30 | 50 | 0 | 33 | 44 | 40 | 45 | 65 | 50 |
| 5 | 0 | 80 | 50 | 50 | 0 | 56 | 40 | 57 | 45 | 50 |
| 3 | 0 | 0 | 13 | 0 | 100 | 48 | 45 | 67 | 55 | 25 |
| 1 | 0 | 25 | 50 | 0 | 0 | 44 | 45 | 55 | 35 | 75 |
| 5 | 22 | 50 | 25 | 0 | 100 | 76 | 45 | 22 | 25 | 75 |
| 5 | 0 | 95 | 100 | 100 | 100 | 76 | 65 | 100 | 70 | 100 |
| 5 | 0 | 95 | 100 | 100 | 100 | 68 | 60 | 78 | 70 | 50 |
| 3 | 0 | 15 | 13 | 50 | 67 | 76 | 60 | 67 | 55 | 25 |
| 2 | 0 | | 38 | | 100 | 60 | 45 | 45 | 25 | 25 |
| 3 | 7.8 | 40 | 38 | | | 56 | 55 | 100 | 60 | 50 |
| 3 | 0 | 5 | 13 | 50 | 33 | 36 | 20 | 67 | 30 | 25 |
| 6 | 0 | 10 | 75 | 25 | 100 | 80 | 50 | 67 | 40 | 25 |
| 5 | 0 | 70 | 63 | 0 | 33 | 48 | 50 | 57 | 40 | 50 |
| 3 | 3.8 | 45 | 100 | | 100 | 84 | 75 | 57 | 60 | |
| 3 | 33.9 | 35 | 25 | 0 | 33 | 32 | 20 | 80 | 20 | 50 |
| 7 | 28 | 95 | 100 | 100 | 100 | 68 | 60 | 90 | 85 | 100 |
| 4 | 3.8 | 30 | 75 | 25 | 100 | 56 | 55 | 55 | 45 | 25 |
| 5 | 14 | 70 | 63 | 25 | 0 | 40 | 40 | 67 | 40 | 75 |
| 3 | 50.54 | 95 | 100 | 100 | 100 | 84 | 85 | 100 | 100 | 50 |
| 3 | 0 | 65 | 88 | 50 | 100 | 72 | 65 | 100 | 70 | 50 |
| 3 | 0 | 75 | 38 | 0 | 0 | 40 | 25 | 45 | 40 | 25 |
| 5 | 0 | 95 | 100 | 100 | 100 | 56 | 60 | 78 | 85 | 75 |
| 7 | 9.8 | 75 | 38 | 0 | 33 | 40 | 40 | 67 | 45 | 75 |
| 5 | 0 | 90 | 75 | 100 | 100 | 76 | 65 | 78 | 60 | 75 |
| 6 | 0 | 0 | 100 | 100 | 100 | 88 | 90 | 80 | 65 | 50 |
| 3 | 0 | 15 | 50 | 50 | 100 | 72 | 50 | 57 | 30 | 25 |
| 3 | 24.5 | 95 | 100 | 100 | 100 | 88 | 90 | 69 | 85 | 50 |
| 7 | 15.75 | 100 | 88 | 100 | 100 | 80 | 70 | 100 | 85 | 50 |
| 5 | 50.33 | 80 | 63 | 0 | 33 | 44 | 45 | 57 | 60 | 50 |
| 5 | 12 | 60 | 88 | 50 | 100 | 64 | 30 | 67 | 35 | 100 |
| 7 | 45.4 | 45 | 25 | 50 | 0 | 28 | 30 | 22 | 35 | 25 |
| 7 | 12 | 50 | 38 | 25 | 100 | 68 | 65 | 80 | 75 | 50 |
| 4 | 0 | 20 | 63 | 75 | 100 | 80 | 35 | 45 | 25 | 25 |
| 5 | 55 | 50 | 13 | 0 | 100 | 84 | 40 | 10 | 90 | 50 |
| 7 | 25.5 | 85 | 50 | 75 | 67 | 56 | 50 | 78 | 60 | 50 |
| 2 | 19.5 | 45 | 75 | 50 | 100 | 68 | 45 | 100 | 55 | 100 |
| 4 | 0 | 50 | 38 | 0 | 100 | 76 | 35 | 45 | 45 | 25 |
| 1 | 24.5 | 40 | 100 | 50 | 100 | 84 | 75 | 90 | 70 | 50 |
| 3 | 11.3 | 55 | 13 | 50 | 0 | 24 | 20 | 80 | 20 | 75 |
| 3 | 14.5 | 20 | 63 | 25 | 100 | 80 | 55 | 100 | | 50 |
| 2 | 21 | 55 | 100 | 75 | 100 | 84 | 80 | 67 | 70 | 50 |
| 5 | 0 | 85 | 88 | 100 | 100 | 80 | 50 | 90 | 70 | 75 |
| 3 | 25.6 | | 38 | 0 | 0 | | | 67 | 40 | |
| 6 | 0 | 65 | 88 | | | 72 | 55 | 55 | 50 | 50 |
| 3 | 6 | 15 | 50 | | | 64 | 50 | 67 | 35 | 25 |
| 5 | 13.6 | 80 | 38 | 25 | 0 | 44 | 40 | 88 | 55 | 50 |
| 5 | 0 | 50 | 38 | 75 | 67 | 72 | 20 | 45 | 40 | 50 |
| 6 | 18.75 | 85 | 100 | 100 | 100 | 84 | 85 | 80 | 80 | 50 |
| 3 | 0 | 65 | 100 | 50 | 33 | 88 | 65 | 100 | 90 | 50 |
| 7 | 0 | 75 | 50 | 0 | 100 | 68 | 40 | 67 | 55 | 100 |
| 3 | 24.5 | 75 | 63 | 50 | 33 | 44 | 30 | 76 | 40 | 50 |
| 7 | 38.2 | 90 | 88 | 100 | 67 | 80 | 80 | 90 | 75 | 50 |
| 3 | 12.25 | 25 | 63 | 25 | 33 | 52 | 55 | 100 | 50 | 50 |
| 7 | 59 | 45 | 88 | 75 | 100 | 76 | 60 | 45 | 55 | 25 |
| 1 | 103.2 | 15 | 75 | 0 | | 60 | 60 | 45 | 40 | 25 |
| 5 | 12 | 60 | 38 | 0 | 0 | 48 | 45 | 57 | 35 | 50 |
| 3 | 0 | 80 | 100 | 100 | 100 | 84 | 65 | 100 | 60 | 100 |
| 7 | 0 | 5 | 25 | 0 | 0 | 40 | 50 | 45 | 80 | 50 |
| 5 | 15.75 | 55 | 25 | 0 | 33 | 28 | 20 | 22 | 85 | 50 |

| | | | | | | | | | | |
|---|---|---|---|---|---|---|---|---|---|---|
| 6 | 0 | 70 | 25 | 25 | 67 | 52 | 35 | 45 | 30 | 75 |
| 3 | 34.5 | 65 | 50 | 0 | 0 | 72 | 40 | 45 | 30 | 25 |
| 2 | 24.7 | 10 | 50 | 0 | 67 | 60 | 30 | 45 | 25 | 25 |
| 5 | 21 | 75 | 63 | 0 | 100 | 84 | 55 | 45 | 65 | 50 |
| 3 | 51.95 | 80 | 75 | 25 | 67 | 52 | 50 | 78 | 60 | 100 |
| 5 | 39.75 | 60 | 50 | 0 | 100 | 56 | 25 | 45 | 50 | 50 |
| 5 | 34 | 60 | 63 | 0 | 100 | 64 | 55 | 22 | 70 | 75 |
| 3 | 53.6 | 25 | 63 | 75 | 100 | 48 | 40 | 45 | 55 | 50 |
| 3 | 64.8 | 70 | 75 | 50 | 100 | 64 | 60 | 100 | 65 | 50 |
| 5 | 61.5 | 85 | 75 | 100 | 100 | 64 | 60 | 80 | 70 | 50 |
| 7 | 58.8 | 75 | 75 | 100 | 100 | 40 | 60 | 90 | 60 | 50 |
| 3 | 14.65 | 75 | 63 | 0 | 0 | 56 | 50 | 45 | 55 | 25 |
| 3 | 14.9 | 75 | 100 | 100 | 100 | 80 | 70 | 67 | 75 | 100 |
| 2 | 0 | 5 | 50 | 25 | 100 | 80 | 65 | 57 | 35 | 25 |
| 3 | 0 | 40 | 50 | | | 44 | 55 | 55 | 50 | 50 |
| 1 | 0 | 10 | 75 | 0 | 33 | 80 | 75 | 78 | 65 | 50 |
| 5 | 0 | 75 | 38 | 50 | 33 | 28 | 35 | 67 | 70 | 50 |
| 4 | 0 | 35 | 75 | 0 | 100 | 76 | 55 | 100 | 40 | 25 |
| 3 | 10.75 | 100 | 88 | 100 | 100 | 56 | 55 | 100 | 90 | 50 |
| 3 | 0 | 45 | 88 | 100 | 100 | 80 | 65 | 90 | 70 | 50 |
| 7 | 0 | 35 | 13 | | | 60 | 40 | 45 | 55 | 50 |
| 5 | 63.8 | 45 | 63 | 50 | 100 | 68 | 65 | 45 | 60 | 25 |
| 3 | 30.05 | 70 | 88 | 100 | 100 | 48 | 80 | 67 | 35 | 25 |
| 3 | 0 | 35 | 63 | 25 | 67 | 64 | 40 | 35 | 55 | 50 |
| 3 | 0 | 35 | 50 | 0 | 0 | 36 | 10 | 45 | 25 | 25 |
| 1 | 11.6 | 50 | 88 | 100 | 100 | 60 | 60 | 100 | 80 | 50 |
| 2 | 0 | 35 | 100 | 25 | 100 | 68 | 40 | 100 | 70 | 50 |
| 5 | 14 | 30 | 63 | 25 | 100 | 76 | 55 | 45 | 65 | 25 |
| 3 | 36.75 | 40 | 100 | | | 84 | 80 | 90 | 95 | 100 |
| 3 | 13.8 | 40 | 88 | 50 | 100 | 84 | 55 | 90 | 70 | 75 |
| 3 | 24 | 50 | 63 | 50 | 33 | 40 | 35 | 51 | 55 | 75 |
| 4 | 0 | 25 | 50 | 0 | 0 | 64 | 40 | 22 | 30 | 25 |
| 5 | 41 | 80 | 63 | 25 | 67 | 52 | 50 | 45 | 60 | 50 |
| 4 | 0 | 10 | 75 | 100 | 33 | 80 | 60 | 100 | 15 | 50 |
| 7 | 0 | 0 | 63 | 0 | 67 | 64 | 40 | 35 | 25 | 0 |
| 5 | 34 | 70 | 63 | 50 | 67 | 56 | 65 | 57 | 75 | 50 |
| 3 | 0 | 85 | 100 | | | 76 | 45 | 100 | 50 | 75 |
| 3 | 0 | 100 | 88 | 100 | 100 | 80 | 75 | 100 | 70 | 50 |
| 1 | 0 | 5 | 50 | 0 | | 72 | 55 | 80 | 45 | 50 |
| 2 | 0 | 0 | 63 | 0 | 100 | 68 | 40 | 45 | 20 | 0 |
| 1 | 0 | 10 | 100 | 75 | 100 | 88 | 80 | 57 | 70 | 25 |
| 3 | 7.8 | 40 | 100 | 100 | 100 | 88 | 80 | 100 | 85 | 75 |
| 5 | 29.75 | 45 | 63 | 75 | 33 | 44 | 35 | 57 | 45 | 75 |
| 3 | 0 | 40 | 50 | 50 | 100 | 56 | 50 | 45 | 65 | 100 |
| 6 | 0 | 0 | 25 | 25 | 100 | 68 | 35 | 67 | 25 | 25 |
| 7 | 7.3 | 35 | 88 | 50 | 100 | 72 | 65 | 100 | 80 | 50 |
| 5 | 0 | 65 | 63 | 50 | 100 | 64 | 40 | 57 | 65 | 75 |
| 5 | 0 | 20 | 75 | 0 | 0 | 60 | 50 | 33 | 50 | 0 |
| 3 | 0 | 80 | 63 | 100 | 100 | 80 | 65 | 80 | 35 | 50 |
| 1 | 10.5 | 25 | 100 | 25 | 100 | 84 | 60 | 20 | 50 | 75 |
| 5 | 29.5 | 95 | 100 | 100 | 100 | 68 | 55 | 100 | 55 | 100 |
| 5 | 0 | 70 | 88 | 50 | 100 | 72 | 60 | 67 | 45 | 50 |
| 2 | 66.93 | 80 | 50 | 0 | 33 | 48 | 50 | 45 | 70 | 100 |
| 3 | 0 | 5 | 38 | 0 | 100 | 76 | 45 | 78 | 85 | 75 |
| 5 | 0 | 100 | 100 | 100 | 100 | 72 | 75 | 80 | 70 | 50 |
| 3 | 72.5 | 65 | 75 | 0 | 100 | 56 | 35 | 67 | 45 | 75 |
| 3 | 0 | 40 | 50 | 50 | 0 | 40 | 45 | 78 | 40 | 50 |
| 4 | 0 | 0 | 63 | 75 | 100 | 60 | 50 | 57 | 40 | 50 |
| 5 | 0 | | 100 | | | | | 45 | 15 | 25 |

| | | | | | | | | | | |
|---|---|---|---|---|---|---|---|---|---|---|
| 3 | 3.5 | 10 | 88 | | 100 | 60 | 65 | 22 | 65 | 75 |
| 7 | 63.6 | 90 | 88 | 100 | 100 | 88 | 85 | 78 | 80 | 75 |
| 4 | 0 | 20 | 38 | | | 48 | 20 | 33 | 50 | 25 |
| 3 | 0 | 75 | 75 | 100 | 100 | 60 | 60 | 100 | 50 | 50 |
| 7 | 0 | 20 | 63 | | 100 | 88 | 65 | 67 | 55 | 50 |
| 3 | 5.8 | 30 | 50 | 0 | 0 | 36 | 40 | 55 | 50 | 25 |
| 7 | 39.2 | 70 | 75 | 50 | 100 | 84 | 65 | 67 | 85 | 50 |
| 3 | 38.75 | 90 | 63 | 50 | 0 | 64 | 50 | 67 | 55 | 100 |
| 6 | 59 | 85 | 75 | 0 | 33 | 68 | 40 | 100 | 60 | 50 |
| 3 | 12.75 | 50 | 38 | 0 | 67 | 64 | 50 | 67 | 55 | 75 |
| 6 | 0 | 5 | 63 | 0 | 0 | 48 | 35 | 33 | 30 | 25 |
| 5 | 0 | 75 | 100 | 100 | 100 | 88 | 80 | 90 | 95 | 50 |
| 2 | 5.8 | 5 | 50 | 0 | 0 | 76 | 55 | 57 | 45 | 100 |
| 2 | 0 | 5 | 100 | 100 | 100 | 88 | 85 | 69 | 80 | |
| 5 | 69 | 65 | 63 | 50 | 100 | 68 | 60 | 57 | 75 | 50 |
| 4 | 9.3 | 5 | 50 | 0 | 100 | 72 | 55 | 45 | 20 | 25 |
| 6 | 0 | 15 | 100 | 25 | 100 | 88 | 65 | 10 | 70 | 75 |
| 2 | 3.9 | 10 | 63 | | 100 | 80 | 65 | 33 | 60 | 50 |
| 5 | 64.75 | 50 | 75 | 0 | 100 | 88 | 50 | 45 | 60 | 50 |
| 5 | 0 | 35 | 13 | 0 | 100 | 72 | 45 | 10 | 80 | 50 |
| 3 | 34.5 | 50 | 100 | 100 | 100 | 84 | 80 | 100 | 85 | 50 |
| 3 | 0 | 85 | 88 | 100 | 100 | 68 | 55 | 100 | 90 | 50 |
| 3 | 18.5 | 100 | 88 | 75 | 100 | 64 | 50 | 67 | 55 | 75 |
| 2 | 7.5 | 70 | 100 | 100 | 100 | 80 | 80 | 100 | 60 | 50 |
| 3 | 21.5 | 100 | 88 | 100 | 100 | 64 | 60 | 100 | 90 | 50 |
| 7 | 0 | 40 | 63 | 25 | 100 | 56 | 35 | 35 | 30 | 50 |
| 6 | 0 | 45 | 88 | 100 | 100 | 72 | 65 | 90 | 70 | 75 |
| 3 | 18.5 | 70 | 63 | 100 | 67 | 48 | 50 | 100 | 80 | 25 |
| 5 | 19.75 | 90 | 75 | 25 | 100 | 72 | 55 | 45 | 75 | 100 |
| 3 | 40.8 | 80 | 75 | 0 | 0 | 68 | 60 | 100 | 40 | 50 |
| 5 | 7 | 75 | 100 | 75 | 67 | 60 | 55 | 57 | 60 | 50 |
| 3 | 18.04 | 100 | 100 | 50 | 100 | 84 | 70 | 100 | 85 | 75 |
| 5 | 0 | 75 | 75 | 50 | 33 | 72 | 60 | 78 | 35 | 100 |
| 7 | 0 | 80 | 100 | 100 | 100 | 80 | 55 | 80 | 70 | 50 |
| 7 | 0 | 85 | 100 | 50 | 33 | 64 | 50 | 67 | 70 | 100 |
| 3 | 0 | 95 | 63 | 25 | 0 | 40 | 30 | 67 | 40 | 50 |
| 3 | 0 | 90 | 63 | 25 | 67 | 56 | 55 | 100 | 60 | 25 |
| 5 | 0 | 50 | 63 | 25 | 100 | 56 | 50 | 78 | 50 | 75 |
| 5 | 37.5 | 70 | 63 | 25 | 67 | 72 | 75 | 57 | 35 | 50 |
| 5 | 75 | 100 | 50 | 100 | 100 | 80 | 40 | 57 | 95 | 0 |
| 5 | 0 | | 50 | | | 44 | 25 | 45 | 50 | 50 |
| 3 | 15 | 80 | 88 | 100 | 100 | 64 | 65 | 100 | 85 | 75 |
| 3 | 4.8 | 15 | 63 | 50 | 100 | 68 | 45 | 45 | 40 | 50 |
| 3 | 0 | 0 | 88 | | | 64 | 55 | 100 | 50 | 50 |
| 6 | 0 | | 50 | 0 | 0 | | | 33 | 40 | 25 |
| 3 | 7.6 | 5 | 25 | 0 | | 64 | 35 | 60 | 20 | 0 |
| 3 | 52.5 | 85 | 25 | 25 | 0 | 36 | 45 | 22 | 75 | 50 |
| 3 | 5.8 | 10 | 75 | 0 | 33 | 68 | 65 | 90 | 60 | 50 |
| 5 | 48 | 90 | 100 | 100 | 100 | 84 | 70 | 100 | 95 | 50 |
| 5 | 0 | 35 | 50 | 0 | 33 | 48 | 25 | 45 | 25 | 50 |
| 3 | 7 | 25 | 38 | 0 | 0 | 56 | 35 | 45 | 30 | 50 |
| 3 | 0 | 0 | 13 | | | 24 | 30 | 45 | 15 | 50 |
| 3 | 3.8 | 0 | 25 | 25 | 100 | 80 | 65 | 10 | 65 | |
| 3 | 11.8 | 40 | 100 | 0 | 0 | 80 | 70 | 100 | 75 | 50 |
| 1 | 0 | 70 | 75 | | | 84 | 75 | 100 | 55 | 50 |
| 5 | 78.6 | 100 | 75 | | 100 | 72 | 70 | 78 | 65 | 75 |
| 5 | 0 | 10 | 38 | 100 | 100 | 44 | 20 | 35 | 15 | 50 |
| 3 | 0 | 50 | 63 | 100 | 100 | 48 | 40 | 55 | 60 | 50 |
| 5 | 0 | 70 | 63 | 25 | 100 | 84 | 65 | 22 | 85 | 50 |

| | | | | | | | | | | |
|---|---|---|---|---|---|---|---|---|---|---|
| 5 | 19.6 | 35 | 38 | 0 | 0 | 52 | 40 | 55 | 50 | 50 |
| 3 | 0 | 0 | 0 | 0 | 0 | 60 | 60 | 100 | 70 | 50 |
| 4 | 0 | 15 | 38 | | 0 | 52 | 40 | 33 | 65 | 25 |
| 3 | 0 | 55 | 75 | 50 | 100 | 64 | 65 | 78 | 30 | 50 |
| 5 | 23 | 90 | 100 | 75 | 100 | 76 | 55 | 90 | 70 | 100 |
| 5 | 0 | 20 | 63 | 0 | 100 | 64 | 30 | 0 | 20 | 0 |
| 3 | 0 | 60 | 88 | 50 | 67 | 84 | 25 | 100 | 40 | 25 |
| 3 | 0 | 95 | 50 | 50 | 0 | 36 | 30 | 100 | 55 | 50 |
| 6 | 0 | 0 | 63 | 50 | 100 | 64 | 55 | 90 | 25 | 50 |
| 5 | 55 | 60 | 38 | 0 | 100 | 72 | 25 | 55 | 50 | 75 |
| 4 | 9.89 | 5 | 50 | 50 | 33 | 68 | 40 | 45 | 60 | 50 |
| 7 | 19.3 | 80 | 100 | 100 | 100 | 84 | 70 | 67 | 75 | 50 |
| 5 | 0 | 50 | 100 | 25 | 33 | 76 | 55 | 45 | 60 | 50 |
| 1 | 0 | 25 | 63 | 0 | 67 | 64 | 40 | 67 | 35 | 50 |
| 5 | 10.4 | 90 | 100 | 100 | 100 | 84 | 60 | 100 | 85 | 50 |
| 4 | 0 | 10 | 100 | 100 | 100 | 88 | 65 | 100 | 75 | 50 |
| 3 | 22.15 | 20 | 0 | 0 | 100 | 64 | 40 | 55 | 30 | 50 |
| 7 | 0 | 0 | 63 | 75 | 100 | 72 | 55 | 35 | 25 | 50 |
| 3 | 0 | 0 | 50 | 0 | | 48 | 20 | 100 | 40 | 25 |
| 3 | 30.5 | 85 | 63 | 75 | 100 | 76 | 70 | 78 | 55 | 50 |
| 5 | 19 | 90 | 100 | 75 | 67 | 76 | 65 | 78 | 75 | 50 |
| 3 | 32 | 90 | 25 | 75 | 0 | 56 | 80 | 100 | 70 | 50 |
| 5 | 18.3 | 95 | 75 | 100 | 100 | 68 | 60 | 80 | 95 | 75 |
| 6 | 7.6 | 80 | 88 | 100 | 100 | 80 | 70 | 88 | 70 | 50 |
| 4 | 7.8 | 40 | 75 | 0 | 100 | 60 | 40 | 67 | 20 | 25 |
| 3 | 0 | 65 | 100 | 50 | 67 | 72 | 65 | 57 | 65 | 25 |
| 5 | 24.5 | 55 | 25 | 0 | 0 | 60 | 40 | 10 | 35 | 0 |
| 2 | 14 | 75 | 88 | 0 | 100 | 76 | 70 | 80 | 75 | 50 |
| 7 | 0 | 85 | 100 | 75 | 100 | 72 | 60 | 67 | 30 | 25 |
| 5 | 0 | 70 | 88 | 0 | 100 | 80 | 55 | 57 | 50 | 75 |
| 7 | 42.4 | 100 | 100 | 100 | 100 | 88 | 90 | 100 | 100 | 50 |
| 3 | 4.3 | 50 | 100 | 0 | 100 | 72 | 60 | 35 | 75 | 50 |
| 3 | 0 | 45 | 88 | 75 | 100 | 68 | 50 | 100 | 45 | 50 |
| 3 | 0 | 0 | 0 | | | 72 | 30 | 60 | 20 | 50 |
| 3 | 24.5 | 45 | 50 | 50 | 33 | 56 | 35 | 45 | 30 | 25 |
| 3 | 15 | 90 | 63 | 75 | 0 | 40 | 45 | 78 | 60 | 50 |
| 5 | 6 | 80 | 25 | 0 | 0 | 36 | 45 | 22 | 35 | 50 |
| 3 | 0 | 75 | 100 | 50 | 100 | 72 | 50 | 67 | 65 | 50 |
| 5 | 0 | 45 | 38 | 25 | 67 | 80 | 45 | 45 | 55 | 50 |
| 5 | 44.5 | 90 | 88 | 0 | 100 | 88 | 55 | 45 | 40 | 50 |
| 1 | 21.6 | 50 | 75 | 75 | 100 | 80 | 80 | 76 | 70 | 50 |
| 4 | 0 | 0 | 38 | 0 | 0 | 48 | 50 | 45 | 25 | 25 |
| 1 | 86 | 80 | 100 | 100 | 100 | 76 | 75 | 80 | 90 | 50 |
| 1 | 0 | 60 | 100 | 25 | 100 | 88 | 50 | 100 | 75 | 50 |
| 5 | 25.25 | 80 | 25 | 0 | 0 | 64 | 75 | 45 | 50 | 100 |
| 1 | 5.8 | 30 | 63 | 100 | 100 | 68 | 65 | 67 | 55 | 50 |
| 7 | 14 | 80 | 88 | 100 | 100 | 68 | 55 | 90 | 40 | 50 |
| 6 | 36 | 0 | 50 | 100 | 33 | 60 | 55 | 45 | 55 | 50 |
| 5 | 0 | 45 | 75 | | | 68 | 40 | 55 | 45 | 25 |
| 3 | 0 | 25 | 88 | 50 | 67 | 68 | 75 | 100 | 90 | |
| 6 | 0 | 50 | 88 | 75 | 100 | 80 | 75 | 100 | 75 | 50 |
| 5 | 24.5 | 70 | 63 | 0 | 100 | 60 | 40 | 45 | 40 | 25 |
| 6 | 23.45 | 70 | 25 | 0 | 100 | 40 | 35 | 45 | 30 | 100 |
| 4 | 10.75 | 25 | 88 | 50 | 100 | 84 | 60 | 67 | 65 | 50 |
| 3 | 100.1 | 100 | 100 | 50 | 67 | 84 | 65 | 100 | 100 | 100 |
| 3 | 119.4 | 100 | 100 | 100 | 100 | 84 | 85 | 100 | 100 | 100 |
| 7 | 25.8 | 90 | 100 | 100 | 100 | 72 | 75 | 80 | 95 | 100 |
| 7 | 3.75 | 50 | 100 | 75 | 100 | 80 | 70 | 80 | 75 | 50 |
| 5 | 9 | 80 | 100 | 50 | 100 | 64 | 45 | 67 | 90 | 50 |

| | | | | | | | | | | |
|---|---|---|---|---|---|---|---|---|---|---|
| 3 | 0 | 50 | 75 | 100 | 100 | 68 | 65 | 90 | 70 | 50 |
| 3 | 27.8 | 70 | 88 | 100 | 100 | 80 | 75 | 100 | 70 | 50 |
| 3 | 6.9 | 15 | 63 | 25 | 100 | 76 | 50 | 57 | 35 | 50 |
| 2 | 0 | 25 | 75 | 100 | 100 | 80 | 65 | 67 | 60 | 100 |
| 3 | 0 | 75 | 13 | | | 64 | 40 | 43 | 55 | 75 |
| 5 | 49 | 90 | 88 | 100 | 100 | 80 | 45 | 90 | 80 | 75 |
| 7 | 16.3 | 95 | 100 | 100 | 100 | 76 | 65 | 100 | 85 | 75 |
| 3 | 0 | 5 | 50 | | 33 | 20 | 40 | 55 | 5 | 75 |
| 3 | 0 | 0 | 50 | | | 48 | 40 | 100 | 25 | 0 |
| 7 | 0 | 40 | 25 | 0 | 0 | 56 | 45 | 45 | 45 | 75 |
| 5 | | 70 | 63 | 0 | 100 | 60 | 30 | 55 | 60 | 50 |
| 6 | 35.2 | 45 | 50 | 0 | 100 | 72 | 55 | 67 | 65 | 75 |
| 7 | 18 | 30 | 75 | | | 52 | 45 | 67 | 45 | 50 |
| 5 | 64.6 | 70 | 88 | 100 | 100 | 68 | 60 | 67 | 60 | 100 |
| 3 | 7 | 15 | 38 | 75 | 100 | 64 | 30 | 35 | 45 | 25 |
| 6 | 31.4 | 95 | 75 | 100 | 100 | 72 | 70 | 100 | 65 | 50 |
| 5 | 0 | 70 | 88 | 50 | 100 | 76 | 65 | 67 | 85 | 75 |
| 6 | 0 | 10 | 75 | | | 64 | 40 | 20 | 55 | 0 |
| 6 | 45 | 55 | 100 | 50 | 100 | 88 | 85 | 80 | 50 | 50 |
| 7 | 0 | 5 | 0 | 25 | 0 | 24 | 35 | 10 | 25 | 0 |
| 5 | 0 | 35 | 88 | 100 | 100 | 88 | 80 | 69 | 50 | 50 |
| 7 | 26 | 80 | 100 | 75 | 100 | 76 | 80 | 100 | 70 | 75 |
| 4 | 61.6 | 55 | 63 | 75 | 33 | 64 | 70 | 100 | 70 | 100 |
| 7 | 33.25 | 55 | 50 | 0 | 100 | 76 | 45 | 45 | 65 | 50 |
| 7 | 78.3 | 100 | 88 | 100 | 100 | 68 | 75 | 100 | 95 | 75 |
| 7 | 7.25 | 85 | 100 | 100 | 100 | 64 | 55 | 57 | 65 | 75 |
| 5 | 4 | 85 | 100 | 75 | 100 | 72 | 65 | 57 | 70 | 75 |
| 7 | 138.6 | 100 | 75 | 50 | 100 | 80 | 75 | 100 | 80 | 75 |
| 3 | 0 | 15 | 50 | 0 | 0 | 44 | 45 | 67 | 55 | 25 |
| 5 | 0 | 45 | 100 | 25 | 100 | 80 | 40 | 45 | 55 | 50 |
| 5 | 35 | 55 | 50 | 50 | 0 | 44 | 30 | 57 | 35 | 75 |
| 4 | 0 | 30 | 88 | 100 | 100 | 80 | 65 | 100 | 75 | 100 |
| 6 | 0 | 0 | 88 | 75 | 100 | 64 | 60 | 80 | 35 | 50 |
| 3 | 0 | 30 | 63 | 75 | 100 | 56 | 55 | 100 | 40 | 100 |
| 1 | 0 | 0 | 25 | 0 | 0 | 48 | 30 | 22 | 40 | 50 |
| 3 | 8.7 | 10 | 38 | 50 | 0 | 32 | 25 | 100 | 85 | 50 |
| 2 | 59.5 | 35 | 88 | 100 | 100 | 68 | 70 | 90 | 85 | 100 |
| 3 | 118.8 | 70 | 100 | 25 | 33 | 68 | 45 | 100 | 75 | 50 |
| 5 | 29.75 | 75 | 75 | 50 | 0 | 32 | 35 | 35 | 30 | 75 |
| 3 | 0 | 50 | 88 | | | 60 | 70 | 100 | 60 | 25 |
| 3 | 0 | 0 | 0 | 0 | 0 | 72 | 55 | 22 | | 25 |
| 3 | 24.5 | 45 | 0 | 0 | 0 | 64 | 30 | 33 | 30 | 25 |
| 5 | 33 | 60 | 38 | 0 | 0 | 36 | 30 | 35 | 30 | 25 |
| 6 | 12 | 5 | 50 | 0 | 0 | 68 | 60 | 88 | 35 | 0 |
| 7 | 0 | 65 | 100 | 25 | 100 | 80 | 70 | 90 | 60 | 50 |
| 1 | 0 | 75 | 50 | 25 | 100 | 80 | 55 | 22 | 60 | 50 |
| 2 | 0 | 5 | 38 | 0 | 100 | 68 | 45 | 57 | 40 | 50 |
| 3 | 32.5 | 70 | 63 | 25 | 100 | 76 | 55 | 45 | 50 | 75 |
| 5 | 0 | 5 | 38 | 0 | 0 | 68 | 70 | 45 | 45 | 25 |
| 5 | 51.6 | 90 | 100 | 100 | 100 | 84 | 80 | 67 | 70 | 75 |
| 1 | 43.3 | 15 | 38 | 100 | 100 | 80 | 80 | 100 | 75 | 75 |
| 5 | 60 | 85 | 100 | 75 | 100 | 80 | 40 | 78 | 60 | 75 |
| 3 | 0 | 25 | 25 | | | 40 | 10 | 33 | | 25 |
| 2 | 0 | 0 | 100 | 100 | | 72 | 60 | 100 | 50 | 50 |
| 2 | 0 | 95 | 100 | 75 | 100 | 80 | 70 | 100 | 75 | 50 |
| 4 | 0 | 45 | 100 | 50 | 100 | 64 | 45 | 67 | 25 | 25 |
| 6 | 16 | 45 | 63 | 75 | 67 | 52 | 45 | 45 | 40 | 25 |
| 2 | 24.5 | 70 | 38 | 25 | 0 | 60 | 40 | 55 | 30 | 75 |
| 5 | 17 | 80 | 63 | 25 | 100 | 64 | 45 | 57 | 65 | 50 |

| | | | | | | | | | | |
|---|---|---|---|---|---|---|---|---|---|---|
| 7 | 18.5 | 40 | 50 | 0 | 100 | 56 | 50 | 45 | 35 | 25 |
| 3 | 28 | 60 | 75 | 50 | 0 | 52 | 60 | 78 | 50 | 100 |
| 5 | 15 | 80 | 63 | 100 | 100 | 56 | 55 | 45 | 55 | 75 |
| 7 | 35.8 | 70 | 63 | 0 | 100 | 64 | 55 | 67 | 75 | 100 |
| 3 | 0 | 5 | 38 | | | 52 | 40 | 65 | 40 | 25 |
| 2 | 70.5 | 40 | 38 | 75 | 100 | 60 | 60 | 90 | 65 | 50 |
| 3 | 0 | 100 | 100 | 100 | 100 | 88 | 60 | 100 | 95 | 100 |
| 3 | 7.5 | 50 | 63 | 0 | 100 | 64 | 40 | 90 | 75 | 50 |
| 2 | 0 | 0 | 75 | | | 72 | 55 | 80 | 50 | 50 |
| 5 | 0 | 75 | 100 | 100 | 100 | 56 | 30 | 57 | 45 | 75 |
| 3 | 28.9 | 75 | 100 | 25 | 100 | 72 | 60 | 100 | 60 | 75 |
| 2 | 25.8 | 5 | 50 | 50 | 67 | 52 | 35 | 67 | 20 | 50 |
| 7 | 62 | 95 | 100 | 100 | 100 | 68 | 70 | 90 | 55 | 50 |
| 6 | 23.9 | 40 | 100 | 50 | 100 | 72 | 55 | 67 | 55 | 50 |
| 3 | 51.85 | 10 | 63 | 100 | 67 | 40 | 45 | 55 | 65 | 25 |
| 3 | 0 | 0 | 13 | 0 | 100 | 84 | 50 | 67 | 20 | 25 |
| 5 | 49 | 70 | 75 | 75 | 100 | 80 | 75 | 67 | 65 | 50 |
| 1 | 28 | 35 | 38 | | | 76 | 55 | 10 | 75 | 25 |
| 4 | 3.8 | 55 | 50 | 50 | 67 | 68 | 45 | 45 | 10 | 25 |
| 5 | 0 | 95 | 100 | 100 | 100 | 72 | 80 | 90 | 80 | 50 |
| 6 | 0 | 15 | 75 | 0 | 100 | 64 | 65 | 45 | 35 | 50 |
| 3 | 40.75 | 70 | 75 | 75 | 100 | 68 | 50 | 67 | 70 | 100 |
| 5 | 21.63 | 85 | 75 | 0 | 100 | 68 | 65 | 57 | 70 | 75 |
| 3 | 0 | 65 | 38 | 0 | 33 | 36 | 40 | 33 | 35 | 25 |
| 2 | 5.8 | 5 | 100 | 100 | 100 | 84 | 85 | 90 | 65 | 100 |
| 5 | 0 | 70 | 63 | 25 | 0 | 56 | 45 | 45 | 40 | 25 |
| 5 | 0 | 35 | 25 | 0 | 100 | 76 | 30 | 22 | 20 | 25 |
| 5 | 11 | 25 | 50 | 0 | 0 | 44 | 45 | 33 | 20 | 50 |
| 3 | 0 | 0 | 25 | 0 | 0 | 52 | 50 | 22 | 50 | 50 |
| 3 | 0 | 15 | 13 | 0 | 0 | 24 | 15 | 0 | 75 | 50 |
| 1 | 0 | 5 | 0 | 25 | 67 | 64 | 55 | 33 | 10 | 25 |
| 3 | 16.38 | 70 | 75 | 0 | 0 | 60 | 40 | 100 | 55 | 50 |
| 5 | 0 | 90 | 88 | 0 | 100 | 64 | 55 | 57 | 65 | 50 |
| 6 | 0 | 60 | 50 | 0 | 0 | 40 | 25 | 100 | 30 | 50 |
| 5 | 10.3 | 80 | 63 | 50 | 100 | 88 | 55 | 45 | 30 | 50 |
| 3 | 12 | 0 | 13 | 0 | 0 | 44 | 35 | 0 | 30 | 0 |
| 2 | 5.7 | 0 | 38 | | | 76 | 60 | 57 | 35 | 25 |
| 6 | 22.15 | 15 | 63 | 0 | 100 | 72 | 50 | 67 | 35 | 25 |
| 2 | 0 | 5 | 63 | | 100 | 72 | 35 | 100 | 80 | 50 |
| 3 | 0 | 60 | 50 | 25 | 0 | 52 | 25 | 100 | 30 | 50 |
| 3 | 30 | 25 | 75 | 25 | 33 | 80 | 75 | 80 | 95 | 50 |
| 5 | 16.5 | 50 | 25 | 0 | 33 | 36 | 45 | 22 | 45 | 25 |
| 2 | 79.6 | 90 | 100 | 25 | 100 | 80 | 70 | 100 | 70 | 25 |
| 5 | 18 | 95 | 100 | 75 | 100 | 60 | 60 | 80 | 75 | 50 |
| 5 | 18 | 65 | 63 | 50 | 100 | 68 | 40 | 57 | 65 | 50 |
| 5 | 0 | 100 | 100 | 100 | 100 | 80 | 85 | 100 | 100 | 75 |
| 5 | 7.6 | 20 | 38 | 0 | 100 | 80 | 30 | 22 | 35 | 50 |
| 3 | 6 | 5 | 75 | 50 | 0 | 44 | 50 | 100 | 90 | 50 |
| 3 | 17.5 | 55 | 50 | 0 | 0 | 60 | 45 | 45 | 50 | 50 |
| 6 | 19.6 | 25 | 88 | | | 80 | 65 | 67 | 70 | 50 |
| 2 | 7.52 | 10 | 75 | 75 | 67 | 72 | 60 | 69 | 45 | 50 |
| 5 | 0 | 70 | 75 | 75 | 100 | 68 | 55 | 45 | 80 | 50 |
| 5 | 8.75 | 50 | 88 | | | 68 | 55 | 45 | 70 | 50 |
| 3 | 25 | 5 | 38 | 25 | 100 | 72 | 45 | 100 | 45 | 75 |
| 3 | 0 | 85 | 63 | 75 | 0 | 48 | 40 | 78 | 60 | 75 |
| 5 | 4.3 | 45 | 50 | 0 | 33 | 56 | 50 | 45 | 75 | 75 |
| 5 | 55.6 | 55 | 25 | 100 | 100 | 28 | 30 | 100 | 55 | 100 |
| 3 | 0 | 5 | 0 | 0 | 0 | 40 | 20 | 100 | 40 | 0 |
| 6 | 0 | 40 | 75 | 0 | 100 | 84 | 75 | 78 | 65 | 50 |

| | | | | | | | | | | |
|---|---|---|---|---|---|---|---|---|---|---|
| 3 | 6 | 70 | 63 | 50 | 100 | 72 | 60 | 57 | 50 | 50 |
| 5 | 5.25 | 55 | 63 | 0 | 0 | 44 | 25 | 47 | 70 | 50 |
| 5 | 84.6 | 50 | 75 | 0 | 67 | 64 | 50 | 67 | 65 | 50 |
| 2 | 78.5 | 65 | 63 | 0 | 100 | 64 | 50 | 45 | 45 | 75 |
| 3 | 5.4 | 25 | 50 | | 67 | 72 | 40 | 100 | 80 | 50 |
| 7 | 96 | 90 | 100 | 75 | 67 | 44 | 35 | 45 | 70 | 75 |
| 7 | 0 | 75 | 100 | 100 | 100 | 84 | 60 | 100 | 90 | 100 |
| 4 | 0 | 0 | 25 | | 0 | 64 | 35 | 80 | 60 | 50 |
| 7 | 34.6 | 100 | 88 | 100 | 100 | 68 | 75 | 100 | 75 | 50 |
| 2 | 5.7 | 70 | 25 | 0 | 33 | 60 | 30 | 55 | 10 | 0 |
| 7 | 15 | 55 | 75 | 75 | 100 | 72 | 70 | 55 | 65 | 100 |
| 2 | 0 | 20 | 38 | 25 | 33 | 56 | 55 | 78 | 65 | 100 |
| 5 | 30 | 100 | 63 | 100 | 33 | 72 | 65 | 100 | 100 | 50 |
| 7 | 15 | 85 | 75 | 25 | 100 | 72 | 55 | 67 | 80 | 50 |
| 3 | 28.6 | 75 | 13 | 0 | 0 | 20 | 35 | 45 | 40 | 25 |
| 5 | 15.6 | 80 | 100 | 100 | 100 | 88 | 70 | 78 | 90 | 100 |
| 6 | 0 | 5 | 50 | 25 | 67 | 76 | 65 | 57 | 55 | 50 |
| 5 | 55 | 40 | 63 | 0 | 100 | 64 | 55 | 45 | 50 | 25 |
| 4 | 0 | 90 | 75 | 50 | 100 | 76 | 70 | 67 | 70 | 75 |
| 7 | 0 | 10 | 38 | 50 | 33 | 76 | 40 | 67 | 40 | 50 |
| 7 | 0 | 20 | 100 | 100 | 100 | 84 | 70 | 45 | 60 | 50 |
| 4 | 0 | 45 | 63 | 100 | 33 | 60 | 60 | 67 | 55 | 75 |
| 6 | 22.2 | 85 | 100 | 100 | 100 | 60 | 70 | 100 | 75 | 50 |
| 5 | 12.7 | 45 | 38 | 25 | 100 | 80 | 65 | 45 | 20 | 50 |
| 3 | 0 | 30 | 50 | 0 | 0 | 56 | 35 | 45 | 50 | 50 |
| 5 | 74.5 | 50 | 63 | 25 | 33 | 56 | 60 | 67 | 50 | 50 |
| 5 | 0 | 5 | 50 | 0 | 33 | 60 | 15 | 22 | 30 | 25 |
| 5 | 23 | 70 | 75 | 50 | 100 | 76 | 60 | 67 | 65 | 50 |
| 4 | 0 | 25 | 63 | 0 | 33 | 60 | 40 | 10 | 30 | 25 |
| 5 | 15.5 | 95 | 100 | 75 | 100 | 72 | 70 | 78 | 80 | 50 |
| 7 | 0 | 90 | 50 | 75 | 100 | 84 | 75 | 90 | 65 | 50 |
| 2 | 0 | 15 | 38 | | | 52 | 50 | 100 | 60 | 50 |
| 6 | 18.5 | 85 | 88 | | 100 | 72 | 75 | 57 | 80 | 100 |
| 3 | 3.5 | 90 | 75 | 50 | 67 | 72 | 55 | 67 | 35 | 50 |
| 5 | 0 | 15 | 25 | 25 | 100 | 72 | 50 | 45 | 40 | 25 |
| 3 | 19.3 | 50 | 63 | 100 | 100 | 56 | 55 | 57 | 70 | 50 |
| 1 | 7 | 25 | 100 | 100 | 100 | 76 | 70 | 100 | 85 | 50 |
| 6 | 12.25 | 50 | 75 | 0 | 100 | 76 | 50 | 22 | 20 | 25 |
| 5 | 0 | 0 | 0 | 0 | 100 | 64 | 20 | 0 | 75 | 50 |
| 4 | 0 | 40 | 100 | 100 | 100 | 80 | 65 | 80 | 25 | 50 |
| 7 | 0 | 80 | 100 | 75 | 100 | 88 | 70 | 78 | 70 | 50 |
| 1 | 0 | 10 | 63 | 0 | 33 | 76 | 65 | 90 | 35 | 50 |
| 5 | 36.1 | 90 | 88 | 0 | 100 | 80 | 60 | 67 | 90 | 50 |
| 4 | 62.4 | 5 | 63 | 0 | 67 | 72 | 60 | 78 | 35 | 100 |
| 2 | 0 | 10 | 38 | 0 | 100 | 76 | 55 | 0 | 20 | 25 |
| 3 | 13.8 | 45 | 63 | 75 | 100 | 52 | 35 | 22 | 65 | 50 |
| 3 | 56.25 | 100 | 50 | 0 | 0 | 76 | 40 | 45 | 90 | 100 |
| 6 | 12 | 80 | 100 | 75 | 100 | 76 | 60 | 35 | 70 | 75 |
| 3 | 67.5 | 100 | 100 | 100 | 100 | 56 | 50 | 100 | 40 | 50 |
| 2 | 0 | 10 | 13 | 0 | 100 | 72 | 50 | 33 | 25 | 25 |
| 7 | 0 | 0 | 50 | | | 72 | 50 | 20 | 30 | 100 |
| 7 | 31.35 | 65 | 50 | 0 | 33 | 68 | 60 | 45 | 60 | 25 |
| 3 | 0 | 35 | 50 | 0 | 0 | 48 | 40 | 47 | 30 | 25 |
| 5 | 52 | 70 | 63 | 100 | 100 | 76 | 65 | 69 | 55 | 25 |
| 6 | 0 | 95 | 88 | 100 | 100 | 76 | 70 | 78 | 60 | 50 |
| 5 | 41.4 | 85 | 75 | | | 56 | 50 | 90 | 60 | 50 |
| 7 | 0 | 40 | 38 | 0 | 0 | 36 | 20 | 45 | 35 | 25 |
| 5 | 23.4 | 95 | 100 | 100 | 67 | 72 | 65 | 78 | 85 | 100 |
| 3 | 0 | 85 | 100 | 100 | 100 | 84 | 65 | 100 | 70 | 50 |

| | | | | | | | | | | |
|---|---|---|---|---|---|---|---|---|---|---|
| 3 | 36.5 | 100 | 50 | 100 | 100 | 64 | 70 | 100 | 65 | 50 |
| 5 | 19.76 | 50 | 63 | 50 | 100 | 76 | 50 | 45 | 65 | 75 |
| 3 | 8.7 | 10 | 38 | | 100 | 80 | 60 | 100 | 100 | 50 |
| 5 | 9 | 35 | 63 | 0 | 100 | 76 | 70 | 45 | 65 | 75 |
| 4 | 39.5 | 60 | 88 | | | 80 | 70 | 67 | 35 | 50 |
| 7 | 0 | 75 | 88 | 75 | 67 | 72 | 50 | 100 | 65 | 75 |
| 3 | 0 | 25 | 63 | | | 52 | 60 | 45 | 30 | 50 |
| 3 | 35.4 | 35 | 0 | 0 | 100 | 52 | 50 | 10 | 45 | 50 |
| 3 | 14.6 | 55 | 75 | 0 | 100 | 56 | 35 | 100 | 40 | 0 |
| 2 | 24 | 15 | 25 | 0 | 100 | 68 | 50 | 90 | 45 | 25 |
| 7 | 0 | 95 | 100 | 100 | 100 | 76 | 70 | 100 | 70 | 50 |
| 7 | 16 | 20 | 63 | 0 | 100 | 72 | 30 | 90 | 35 | 75 |
| 3 | 0 | 55 | 75 | 0 | 0 | 36 | 30 | 45 | 40 | 75 |
| 6 | 0 | 0 | 75 | 50 | 100 | 56 | 60 | 100 | 45 | 75 |
| 3 | 0 | 65 | 75 | | | 68 | 35 | 67 | 70 | 50 |
| 2 | 0 | 0 | 50 | 50 | 100 | 80 | 50 | 78 | 20 | 0 |
| 4 | 7.8 | 15 | 63 | 0 | 0 | 48 | 35 | 67 | 30 | 25 |
| 7 | 24 | 35 | | 0 | 100 | 56 | 40 | 22 | 40 | 25 |
| 5 | 34 | 85 | 75 | 100 | 100 | 72 | 55 | 67 | 65 | 75 |
| 2 | 34.4 | 20 | 75 | 100 | 100 | 80 | 60 | 57 | 60 | 50 |
| 2 | 20.3 | 30 | 100 | 25 | 100 | 64 | 45 | 67 | 40 | 75 |
| 4 | 0 | 0 | 63 | 0 | 100 | 76 | 70 | 100 | 55 | 25 |
| 6 | 57.4 | 30 | 63 | 0 | 0 | 64 | 60 | 45 | 15 | 25 |
| 5 | 0 | 80 | 63 | 0 | 0 | 56 | 50 | 55 | 45 | 50 |
| 3 | 2.5 | 60 | 38 | 0 | 0 | 64 | 25 | 67 | 50 | 25 |
| 5 | 10.63 | 60 | 75 | 50 | 100 | 68 | 65 | 57 | 65 | 50 |
| 5 | 0 | 80 | 100 | 100 | 100 | 80 | 65 | 80 | 50 | 75 |
| 6 | 30.05 | 45 | 100 | 100 | 100 | 88 | 85 | 78 | 90 | 50 |
| 3 | 65.5 | 95 | 75 | 75 | 100 | 56 | 60 | 100 | 80 | 75 |
| 3 | 0 | 25 | 13 | 0 | 0 | 52 | 45 | 43 | 20 | 25 |
| 3 | 28 | 50 | 75 | 25 | 100 | 64 | 65 | 57 | 55 | 50 |
| 6 | 0 | 10 | 88 | 100 | 100 | 64 | 60 | 45 | 30 | |
| 3 | 39.5 | 85 | 50 | 25 | 0 | 72 | 55 | 90 | 70 | 75 |
| 1 | 0 | 10 | 100 | | | 88 | 80 | 69 | 50 | 100 |
| 2 | 0 | 0 | 13 | 75 | 100 | 68 | 30 | 57 | 30 | 25 |
| 3 | 0 | 40 | 50 | 25 | 33 | 72 | 50 | 55 | 70 | 75 |
| 5 | 0 | 95 | 88 | 100 | 100 | 64 | 70 | 100 | 65 | 100 |
| 6 | 16.8 | 80 | 75 | 75 | 100 | 68 | 55 | 67 | 65 | 100 |
| 5 | 0 | 60 | 88 | 75 | 100 | 76 | 50 | 57 | 40 | 50 |
| 5 | 0 | 80 | 88 | 75 | 100 | 64 | 65 | 57 | 20 | 50 |
| 5 | 18 | 100 | 100 | 100 | 100 | 72 | 70 | 100 | 100 | 100 |
| 3 | 31.2 | 85 | 38 | 50 | 33 | 52 | 40 | 45 | 65 | 75 |
| 3 | 12 | 50 | 100 | 100 | 100 | 72 | 80 | 100 | 80 | 50 |
| 5 | 12.9 | 55 | 50 | | 100 | 68 | 40 | 57 | 70 | 50 |
| 3 | 0 | 100 | 88 | 100 | 100 | 72 | 90 | 100 | 75 | 50 |
| 3 | 0 | 0 | 50 | 0 | 0 | 48 | 50 | 53 | 45 | 50 |
| 2 | 0 | 0 | 50 | 0 | 100 | 52 | 35 | 45 | 65 | 75 |
| 5 | 15 | 25 | 38 | 0 | 0 | 36 | 30 | 45 | 30 | 50 |
| 7 | 10 | 55 | 100 | 50 | 100 | 84 | 30 | 57 | 45 | 25 |
| 7 | 0 | 25 | 63 | 50 | 100 | 64 | 35 | 45 | 20 | 50 |
| 5 | 0 | 50 | 75 | 0 | 100 | 72 | 45 | 45 | 55 | 25 |
| 7 | 7.6 | 35 | 63 | 0 | 100 | 84 | 30 | 67 | 25 | 25 |
| 3 | 28.3 | 80 | 63 | 50 | 100 | 60 | 55 | 100 | 40 | 50 |
| 3 | 0 | 55 | 13 | 0 | 100 | 44 | 30 | 78 | 20 | 50 |
| 3 | 20.4 | 25 | 75 | 100 | 100 | 80 | 70 | 67 | 75 | 75 |
| 5 | 48.4 | 90 | 50 | 25 | 0 | 32 | 35 | 45 | 35 | 50 |
| 5 | 0 | 80 | 100 | 100 | 100 | 80 | 60 | 90 | 65 | 100 |
| 3 | 25.8 | 35 | 50 | 0 | 0 | 52 | 35 | 100 | 25 | 75 |
| 3 | 33.5 | 70 | 63 | 25 | 67 | 60 | 55 | 78 | 60 | 50 |

| | | | | | | | | | | |
|---|---|---|---|---|---|---|---|---|---|---|
| 3 | 0 | 100 | 63 | 100 | 0 | 48 | 50 | 100 | 45 | 50 |
| 7 | 28 | 80 | 88 | 100 | 0 | 68 | 70 | 90 | 65 | 100 |
| 5 | 0 | 55 | 25 | 0 | 0 | 60 | 45 | 22 | 45 | 0 |
| 3 | 0 | 5 | 38 | | | 60 | 45 | 45 | 40 | 25 |
| 2 | 24.75 | 5 | 50 | 25 | 67 | 68 | 45 | 45 | 40 | 75 |
| 7 | 37.5 | 75 | 88 | 0 | 67 | 48 | 55 | 67 | 60 | 100 |
| 5 | 63 | 80 | 63 | 50 | 100 | 72 | 45 | 57 | 45 | 25 |
| 4 | 0 | 20 | 25 | 0 | 0 | 48 | 45 | 45 | 35 | 25 |
| 5 | 9.3 | 20 | 13 | 0 | 67 | 36 | 40 | 10 | 10 | 0 |
| 7 | 0 | 60 | 88 | 25 | 33 | 68 | 60 | 100 | 85 | 75 |
| 3 | 4.3 | 10 | 38 | | | 88 | 80 | 100 | 85 | 50 |
| 5 | 24.75 | 90 | 75 | 100 | 100 | 72 | 50 | 67 | 60 | 75 |
| 3 | 0 | 70 | 88 | 50 | 0 | 68 | 35 | 100 | 25 | 75 |
| 5 | 14.4 | 10 | 75 | 0 | 100 | 60 | 30 | 22 | 10 | 25 |
| 5 | 0 | 60 | 63 | 0 | 100 | 52 | 40 | 55 | 35 | 50 |
| 5 | 18.05 | 85 | 100 | 75 | 67 | 76 | 50 | 67 | 65 | 50 |
| 1 | 0 | | 38 | 25 | 67 | 68 | 80 | 45 | 50 | 25 |
| 6 | 42.5 | 0 | 100 | 0 | 100 | 88 | 50 | 90 | 30 | 75 |
| 3 | 8.6 | 80 | 75 | 50 | 33 | 76 | 45 | 67 | 50 | 50 |
| 5 | 36 | 10 | 50 | 0 | 67 | 80 | 55 | 22 | 35 | 25 |
| 3 | 0 | 85 | 88 | 100 | 100 | 76 | 70 | 67 | 65 | 50 |
| 7 | 12 | 15 | 63 | 50 | 100 | 60 | 25 | 78 | 35 | 75 |
| 5 | 31.6 | 75 | 75 | 50 | 100 | 76 | 60 | 67 | 80 | 50 |
| 6 | 38 | 35 | 75 | 25 | 33 | 80 | 60 | 100 | 35 | 50 |
| 2 | 26.95 | 25 | 50 | 100 | 100 | 60 | 45 | 57 | 30 | 75 |
| 5 | 0 | 50 | 88 | 100 | 100 | 80 | 65 | 100 | 75 | 100 |
| 3 | 23.6 | 50 | 63 | 75 | 67 | 80 | 35 | 78 | 45 | 50 |
| 2 | 23.4 | 35 | 63 | 25 | 100 | 64 | 55 | 67 | 65 | 75 |
| 3 | 0 | 35 | 75 | 75 | 100 | 56 | 35 | 78 | 20 | 25 |
| 3 | 16.4 | 45 | 75 | 50 | 100 | 60 | 75 | 45 | 25 | 25 |
| 3 | 15.45 | 45 | 75 | 50 | 100 | 60 | 75 | 45 | 25 | 25 |
| 2 | 34.2 | 5 | 88 | 100 | 100 | 72 | 70 | 55 | 40 | 50 |
| 2 | 14.5 | 45 | 63 | 0 | 100 | 68 | 55 | 55 | 50 | 75 |
| 5 | 0 | 70 | 100 | 100 | 100 | 76 | 50 | 57 | 75 | 50 |
| 5 | 0 | 70 | 50 | 75 | 0 | 40 | 35 | 67 | 75 | 50 |
| 5 | 72 | 70 | 63 | 75 | 100 | 56 | 50 | 57 | 50 | 50 |
| 2 | 0 | 0 | 100 | 100 | 100 | 88 | 65 | 80 | 75 | 50 |
| 3 | 22.5 | 70 | 63 | 0 | 100 | 56 | 30 | 67 | 10 | 25 |
| 3 | 0 | 60 | 50 | 25 | 0 | 40 | 50 | 100 | 75 | 50 |
| 3 | 30.3 | 90 | 100 | 100 | 100 | 88 | 70 | 47 | 45 | 50 |
| 6 | 26.25 | 65 | 63 | 0 | 100 | 72 | 65 | 67 | 45 | 50 |
| 4 | 6.45 | 40 | 63 | 25 | 100 | 64 | 60 | 10 | 70 | 50 |
| 3 | 0 | 50 | 100 | 100 | 100 | 88 | 90 | 67 | 90 | 100 |
| 5 | 0 | 65 | 50 | 25 | 100 | 64 | 20 | 45 | 50 | 25 |
| 6 | 0 | 15 | 63 | 0 | 0 | 44 | 45 | 33 | 45 | 25 |
| 3 | 8.1 | 45 | 25 | 0 | 33 | 44 | 20 | 45 | 40 | 50 |
| 2 | 20 | 35 | 100 | 0 | 100 | 88 | 85 | 80 | 70 | 75 |
| 4 | 0 | 60 | 63 | 0 | 100 | 80 | 55 | 100 | 40 | 25 |
| 5 | 41 | 95 | 50 | 75 | 67 | 52 | 45 | 57 | 50 | 50 |
| 4 | 0 | 25 | 38 | 0 | 0 | 48 | 40 | 67 | 65 | 75 |
| 3 | 28.65 | | 88 | 50 | 100 | 76 | 50 | 90 | 70 | 50 |
| 4 | 0 | 5 | 25 | 25 | 33 | 48 | 40 | 78 | 35 | 50 |
| 3 | 0 | 10 | 13 | | | 36 | 25 | 10 | 25 | 0 |
| 2 | 12.9 | 10 | 88 | | | 88 | 70 | 10 | 90 | 50 |
| 1 | 0 | 0 | 13 | | | 40 | 30 | 33 | 10 | 0 |
| 5 | 12 | 75 | 63 | 0 | 100 | 80 | 40 | 45 | 75 | 50 |
| 3 | 54 | 95 | 100 | | 100 | 72 | 55 | 67 | 55 | 50 |
| 3 | 0 | 100 | 100 | 100 | 100 | 84 | 80 | 100 | 70 | 50 |
| 7 | 74.4 | 100 | 25 | 100 | 100 | 48 | 45 | 100 | 70 | 50 |

| | | | | | | | | | | |
|---|---|---|---|---|---|---|---|---|---|---|
| 5 | 4.25 | 75 | 63 | 100 | 33 | 36 | 50 | 67 | 70 | 50 |
| 2 | 30.4 | 55 | 75 | 0 | 67 | 68 | 50 | 100 | 45 | 75 |
| 7 | 12 | 0 | 75 | 100 | 67 | 52 | 65 | 100 | 50 | 25 |
| 6 | 0 | 10 | 100 | 100 | 100 | 76 | 40 | 45 | 20 | 25 |
| 7 | 11.25 | 90 | 100 | 100 | 100 | 76 | 80 | 100 | 55 | 50 |
| 6 | 143.7 | 25 | 63 | 50 | 100 | 68 | 70 | 35 | 50 | 75 |
| 3 | 0 | 25 | 38 | 0 | 0 | 48 | 35 | 22 | 30 | 25 |
| 5 | 29.4 | 75 | 75 | 50 | 100 | 76 | 55 | 67 | 60 | 75 |
| 3 | 0 | 65 | 50 | 0 | 0 | 44 | 30 | 45 | 30 | 50 |
| 2 | 0 | 0 | 63 | 0 | 100 | 76 | 70 | 67 | 55 | 25 |
| 3 | 7.5 | 60 | 38 | 75 | 67 | 60 | 45 | 100 | 45 | 50 |
| 3 | 34.25 | 95 | 100 | 100 | 100 | 76 | 70 | 100 | 100 | 75 |
| 5 | 8.6 | 70 | 50 | 0 | 100 | 68 | 45 | 35 | 50 | 50 |
| 6 | 0 | 10 | 63 | 0 | 0 | 56 | 55 | 55 | 45 | 50 |
| 3 | 0 | 70 | 50 | 100 | 0 | 28 | 30 | 100 | 55 | 50 |
| 3 | 18 | 100 | 75 | 100 | 100 | 60 | 45 | 47 | 65 | 50 |
| 7 | 8.6 | 15 | 25 | 0 | 0 | 32 | 30 | 10 | 50 | 50 |
| 3 | 42 | 85 | 63 | 0 | 100 | 68 | 40 | 45 | 70 | 75 |
| 3 | 23.8 | 100 | 63 | 100 | 0 | 68 | 70 | 100 | 95 | 100 |
| 6 | 16.75 | 80 | 38 | 0 | 0 | 56 | 25 | 45 | 50 | 25 |
| 7 | 0 | 0 | 25 | 0 | 100 | 72 | 25 | 43 | 5 | 50 |
| 5 | 0 | 80 | 100 | 100 | 100 | 68 | 65 | 100 | 80 | 100 |
| 3 | 0 | 40 | 100 | 0 | 100 | 84 | 70 | 100 | 35 | 50 |
| 3 | 0 | 10 | 63 | 0 | 0 | 72 | 75 | 78 | 85 | 50 |
| 5 | 0 | 60 | 88 | 50 | 100 | 88 | 60 | 57 | 65 | 50 |
| 1 | 0 | 10 | 25 | 25 | 67 | 48 | 50 | 100 | 80 | 75 |
| 3 | 11.5 | 95 | 75 | 100 | 100 | 80 | 55 | 55 | 75 | 100 |
| 3 | 33.6 | 0 | 0 | 25 | 0 | 40 | 35 | 100 | 15 | 0 |
| 6 | 25.8 | 5 | 38 | 0 | 100 | 88 | 60 | 57 | 45 | 50 |
| 7 | 0 | 20 | 75 | 25 | 67 | 72 | 40 | 45 | 30 | 50 |
| 5 | 62.3 | 60 | 63 | 0 | 100 | 80 | 60 | 45 | 70 | 25 |
| 3 | 4.3 | 15 | 50 | 50 | 100 | 64 | 40 | 100 | 25 | 50 |
| 1 | 0 | 45 | 63 | 50 | 67 | 72 | 55 | 67 | 65 | 75 |
| 2 | 14 | 15 | 38 | 0 | 0 | 40 | 25 | 22 | 20 | 25 |
| 1 | 33.4 | 55 | 63 | 0 | 100 | 76 | 55 | 67 | 40 | 100 |
| 5 | 31.7 | 75 | 75 | 0 | 100 | 48 | 40 | 55 | 70 | 50 |
| 4 | 0 | 50 | 50 | 0 | 33 | 56 | 35 | 90 | 10 | 0 |
| 3 | 13.6 | 100 | 75 | 100 | 67 | 72 | 70 | 100 | 80 | 50 |
| 5 | 41.7 | | 63 | | 100 | 72 | 60 | 45 | 40 | |
| 7 | 136.4 | 100 | 13 | 100 | 100 | 68 | 80 | 78 | 85 | 75 |
| 2 | 0 | 0 | 25 | 0 | 0 | 44 | 25 | 22 | 45 | 25 |
| 5 | 77.5 | 30 | 50 | 0 | 0 | 60 | 25 | 33 | 45 | 50 |
| 3 | 4.3 | 50 | 63 | 0 | 0 | 72 | 60 | 100 | 50 | 75 |
| 4 | 12 | 5 | 38 | 0 | 100 | 72 | 30 | 33 | 35 | 25 |
| 7 | 0 | 25 | 0 | | | 20 | 25 | 35 | 25 | 25 |
| 7 | 28.5 | 80 | 63 | 0 | 33 | 76 | 50 | 33 | 55 | 25 |
| 5 | 49 | 60 | 88 | 25 | 100 | 84 | 75 | 45 | 60 | 25 |
| 3 | 106 | 65 | 100 | 50 | 100 | 80 | 50 | 67 | 80 | 50 |
| 1 | 0 | 15 | 100 | | | 72 | 65 | 90 | 55 | 50 |
| 5 | 12.2 | 50 | 75 | 0 | 0 | 64 | 40 | 45 | 30 | 0 |
| 7 | 0 | 100 | 50 | 100 | 100 | 80 | 60 | 100 | 80 | 50 |
| 6 | 0 | 95 | 38 | 0 | 0 | 60 | 35 | 45 | 5 | 0 |
| 6 | 0 | 0 | 100 | 100 | 100 | 60 | 60 | 80 | 60 | 25 |
| 5 | 0 | 85 | 75 | 100 | 0 | 56 | 40 | 90 | 70 | 50 |
| 4 | 0 | 0 | 38 | | | 72 | 55 | 100 | 40 | 25 |
| 7 | 0 | 10 | 88 | 0 | 67 | 84 | 65 | 33 | 55 | 50 |
| 5 | 36.5 | 100 | 63 | 25 | 33 | 64 | 40 | 67 | 20 | 50 |
| 3 | 0 | 15 | 0 | 0 | | 72 | 55 | 0 | 15 | 0 |
| 7 | 0 | 10 | 100 | 50 | | 72 | 55 | 0 | 35 | 50 |

| | | | | | | | | | | |
|---|---|---|---|---|---|---|---|---|---|---|
| 3 | 47.4 | 95 | 75 | 50 | 33 | 68 | 65 | 78 | 50 | 25 |
| 3 | 5.5 | 30 | 50 | 0 | 100 | 60 | 45 | 55 | 40 | 25 |
| 1 | 120.4 | 25 | 50 | 100 | 100 | 64 | 60 | 78 | 60 | 100 |
| 3 | 19.8 | 100 | 100 | 100 | 100 | 84 | 75 | 100 | 85 | 50 |
| 5 | 20.7 | 45 | 75 | 25 | 100 | 72 | 65 | 45 | 80 | 25 |
| 7 | 10.5 | 70 | 50 | 0 | 100 | 80 | 55 | 55 | 50 | 50 |
| 5 | 0 | 25 | 75 | 0 | 0 | 64 | 45 | 35 | 45 | 50 |
| 3 | 6.88 | 65 | 25 | 0 | 100 | 68 | 40 | 45 | 60 | 50 |
| 5 | 8.5 | 45 | 88 | 0 | 0 | 84 | 45 | 45 | 40 | 50 |
| 1 | 22.8 | 35 | 63 | 0 | 100 | 76 | 75 | 45 | 75 | 50 |
| 6 | 0 | 10 | 0 | 50 | 100 | 48 | 40 | 22 | 50 | 0 |
| 2 | 0 | 0 | 100 | 25 | 67 | 84 | 70 | 80 | 75 | 25 |
| 5 | 11.3 | 45 | 63 | 50 | 100 | 60 | 50 | 67 | 40 | 50 |
| 3 | 24 | 15 | 88 | | | 56 | 55 | 100 | 35 | 50 |
| 6 | 17.5 | 55 | 100 | 50 | 67 | 72 | 55 | 100 | 55 | 75 |
| 5 | 0 | 70 | 75 | 75 | 100 | 72 | 80 | 57 | 45 | 50 |
| 7 | 11.6 | 80 | 100 | | | 80 | 80 | 100 | 50 | 75 |
| 7 | 29.6 | 75 | 75 | 25 | 100 | 80 | 70 | 78 | 65 | 100 |
| 5 | 27.25 | 55 | 100 | 100 | 100 | 80 | 55 | 67 | 65 | 50 |
| 3 | 0 | 50 | 75 | 25 | 33 | 60 | 55 | 78 | 70 | 50 |
| 5 | 16.8 | 60 | 75 | 25 | 100 | 80 | 40 | 57 | 60 | 75 |
| 5 | 31 | 85 | 100 | 100 | 100 | 80 | 70 | 67 | 95 | 100 |
| 7 | 8 | 90 | 100 | 100 | 100 | 84 | 50 | 100 | 80 | 50 |
| 5 | 4 | 35 | 25 | 0 | 33 | 44 | 35 | 33 | 30 | 25 |
| 5 | 24.5 | 50 | 38 | 75 | 67 | 40 | 40 | 10 | 30 | 25 |
| 7 | 43.3 | 55 | 100 | 25 | 100 | 76 | 40 | 22 | 40 | 50 |
| 5 | 71.5 | 100 | 100 | 100 | 100 | 76 | 70 | 100 | 90 | 100 |
| 4 | 11 | 10 | 38 | 0 | 100 | 64 | 50 | 57 | 30 | 25 |
| 4 | 0 | 65 | 63 | 50 | 67 | 76 | 60 | 100 | 50 | 50 |
| 3 | 0 | 35 | 50 | 0 | 0 | | | 45 | 30 | 25 |
| 5 | 0 | 30 | 50 | 25 | 67 | 48 | 30 | 35 | 25 | 25 |
| 7 | 23.4 | 60 | 100 | 25 | 100 | 80 | 45 | 80 | 90 | 100 |
| 1 | 17.2 | 15 | 25 | 0 | 33 | 28 | 15 | 78 | 5 | 25 |
| 5 | 0 | 45 | 38 | 50 | 100 | 76 | 40 | 45 | 25 | 25 |
| 7 | 35 | 80 | 100 | 100 | 100 | 68 | 65 | 69 | 55 | 50 |
| 2 | 37.05 | 0 | 63 | 25 | 100 | 72 | 50 | 67 | 70 | 75 |
| 3 | 33.1 | 95 | 100 | 75 | 100 | 76 | 65 | 100 | 80 | 75 |
| 3 | 7.8 | 100 | 75 | 25 | 100 | 68 | 65 | 88 | 65 | 50 |
| 5 | 0 | 60 | 63 | 0 | | 72 | 25 | 55 | 45 | 50 |
| 5 | 0 | 55 | 38 | 25 | 100 | 80 | 60 | 33 | 50 | 50 |
| 7 | 0 | 95 | 63 | 25 | 100 | 60 | 45 | 67 | 50 | 75 |
| 3 | 15 | 30 | 100 | 25 | 67 | 80 | 90 | 100 | 85 | 75 |
| 5 | 0 | 80 | 100 | 75 | 100 | 80 | 75 | 78 | 90 | 75 |
| 2 | 0 | 80 | 100 | 100 | 100 | 64 | 55 | 67 | 75 | 75 |
| 5 | 0 | 50 | 25 | 0 | 0 | 48 | 35 | 22 | 20 | 50 |
| 6 | 0 | 0 | 50 | 0 | 100 | 72 | 10 | 55 | 35 | 25 |
| 2 | 0 | | 88 | 100 | 100 | 56 | 65 | 80 | 65 | 50 |
| 6 | 0 | 5 | 38 | 0 | 100 | 84 | 60 | 43 | 60 | 50 |
| 4 | 0 | 50 | 63 | 25 | 100 | 72 | 55 | 57 | 10 | 25 |
| 7 | 32 | 30 | 100 | 50 | 100 | 80 | 55 | 45 | 55 | 75 |
| 3 | 5.5 | 15 | 50 | 0 | | 60 | 70 | 67 | 65 | 50 |
| 7 | 51 | 85 | 88 | 100 | 100 | 72 | 60 | 78 | 65 | 50 |
| 5 | 0 | 100 | 100 | 100 | 100 | 76 | 50 | 90 | 85 | 50 |
| 5 | 0 | 60 | 50 | 75 | 67 | 64 | 35 | 67 | 45 | 75 |
| 5 | 79.2 | 75 | 75 | 25 | 100 | 68 | 65 | 57 | 30 | 50 |
| 5 | 14.6 | 85 | 75 | 75 | 100 | 84 | 70 | 57 | 75 | 50 |
| 7 | 0 | 65 | 63 | 0 | 100 | 84 | 60 | 67 | 45 | 75 |
| 3 | 12.1 | 40 | 75 | 0 | | 56 | 50 | 57 | 60 | 50 |
| 2 | 12 | 5 | 50 | 0 | 0 | 64 | 65 | 45 | 75 | 100 |

| | | | | | | | | | | |
|---|---|---|---|---|---|---|---|---|---|---|
| 7 | 24.2 | 50 | 88 | 25 | 100 | 84 | 90 | 100 | 75 | 50 |
| 3 | 0 | 5 | 13 | 25 | 33 | 24 | 35 | 33 | 15 | 0 |
| 6 | 7.5 | 50 | 50 | 0 | 0 | 64 | 65 | 67 | 50 | 100 |
| 3 | 0 | 65 | 75 | 100 | 100 | 80 | 50 | 100 | 40 | 25 |
| 4 | 0 | 40 | 13 | | | | | 100 | 60 | 0 |
| 5 | 23.5 | 80 | 63 | 25 | 100 | 56 | 55 | 67 | 80 | 50 |
| 3 | 27.2 | 70 | 50 | 0 | 100 | 64 | 35 | 100 | 55 | 50 |
| 4 | 0 | 20 | 75 | 50 | 100 | 60 | 40 | 78 | 50 | 25 |
| 3 | 0 | 40 | 75 | 25 | 100 | 68 | 60 | 100 | 65 | 100 |
| 5 | 75.75 | 30 | 13 | 0 | 0 | 36 | 15 | 33 | 30 | 25 |
| 5 | 0 | 70 | 100 | | 100 | 80 | 75 | 67 | 80 | 50 |
| 5 | 46.38 | 55 | 50 | 100 | 100 | 56 | 50 | 22 | 50 | 75 |
| 7 | 3.75 | 75 | 63 | 75 | 100 | 80 | 55 | 57 | 70 | 50 |
| 6 | 25.8 | 75 | 88 | 0 | 100 | 68 | 45 | 78 | 60 | 100 |
| 3 | 0 | 55 | 50 | 50 | 100 | 76 | 60 | 45 | 55 | 100 |
| 3 | 0 | 25 | 0 | 0 | 100 | 56 | 20 | 0 | 25 | 50 |
| 3 | 20 | 65 | 50 | 0 | 33 | 40 | 40 | 100 | 30 | 25 |
| 5 | 0 | 100 | 38 | 100 | 0 | 32 | 25 | 100 | 55 | 100 |
| 3 | 41.5 | 25 | 50 | 0 | 0 | 72 | 60 | 78 | 50 | 75 |
| 3 | 21.7 | 95 | 63 | 75 | 67 | 56 | 70 | 67 | 60 | 50 |
| 3 | 7.8 | 45 | 38 | 25 | 33 | 56 | 15 | 33 | 25 | 25 |
| 5 | 51.6 | 90 | 25 | 25 | 0 | 44 | 45 | 35 | 45 | 50 |
| 3 | 0 | 30 | 63 | 25 | 33 | 68 | 20 | 33 | 40 | 75 |
| 5 | 20.5 | 70 | 50 | 50 | 33 | 32 | 20 | 47 | 80 | 50 |
| 5 | 30.1 | 100 | 88 | 100 | 100 | 80 | 75 | 100 | 60 | 100 |
| 3 | 0 | 100 | 38 | | | 36 | 25 | 80 | 25 | 0 |
| 3 | 0 | 0 | 63 | 0 | 67 | 60 | 40 | 22 | 50 | 25 |
| 7 | 27.7 | 85 | 88 | 25 | 0 | 68 | 75 | 78 | 80 | 100 |
| 3 | 0 | | 50 | 100 | 0 | 36 | 30 | 55 | 60 | 75 |
| 3 | 12.25 | 100 | 88 | 100 | 100 | 80 | 40 | 100 | 65 | 75 |
| 6 | 4.3 | 10 | 63 | 25 | 67 | 68 | 35 | 10 | 55 | 50 |
| 6 | 24.5 | 80 | 100 | 100 | 100 | 72 | 45 | 90 | 65 | 100 |
| 3 | 27.5 | 95 | 100 | 100 | 67 | 64 | 65 | 90 | 70 | 75 |
| 3 | 0 | | 25 | | | 36 | 15 | 100 | 20 | 25 |
| 1 | 0 | 5 | 13 | 0 | 0 | 40 | 25 | 22 | 25 | 25 |
| 5 | 0 | 90 | 75 | 75 | 33 | 64 | 45 | 78 | 70 | 50 |
| 3 | 18.25 | 95 | 50 | 25 | 67 | 56 | 50 | 78 | 50 | 75 |
| 5 | 32 | 55 | 75 | 0 | 100 | 68 | 45 | 67 | 60 | 50 |
| 5 | 0 | 85 | 100 | 100 | 100 | 80 | 45 | 57 | 50 | 50 |
| 7 | 10.5 | 35 | 50 | 25 | 100 | 48 | 30 | 35 | 10 | 25 |
| 3 | 49 | 75 | 63 | 50 | 100 | 72 | 35 | 55 | 40 | 50 |
| 6 | 0 | 5 | 63 | 50 | 100 | 64 | 55 | 78 | 15 | 25 |
| 2 | 5.8 | 35 | 100 | 100 | 100 | 88 | 65 | 90 | 65 | 100 |
| 4 | 0 | 60 | 25 | 0 | 0 | 64 | 40 | 100 | 65 | 25 |
| 6 | 32.35 | 35 | 38 | 0 | 100 | 56 | 50 | 33 | 35 | 25 |
| 5 | 0 | 55 | 63 | 75 | 0 | 44 | 35 | 67 | 35 | 50 |
| 5 | 63.7 | 70 | 63 | 100 | 100 | 52 | 40 | 67 | 70 | 75 |
| 5 | 0 | 30 | 25 | 0 | 100 | 60 | 25 | 10 | 30 | 25 |
| 3 | 10.5 | 100 | 75 | 100 | 33 | 56 | 60 | 100 | 55 | 25 |
| 6 | 0 | 50 | 100 | 100 | 100 | 80 | 60 | 100 | 75 | 50 |
| 2 | 47.3 | | 13 | 25 | 0 | 16 | 25 | 67 | 55 | 100 |
| 1 | 0 | 0 | 0 | 0 | 0 | 8 | 10 | 0 | 0 | 0 |
| 6 | 0 | 5 | 50 | 25 | 67 | | | 0 | 30 | 50 |
| 3 | 38.6 | 95 | 100 | 100 | 100 | 84 | 85 | 100 | 95 | 50 |
| 5 | 57.8 | 100 | 100 | 100 | 100 | 80 | 80 | 90 | 85 | 75 |
| 5 | 0 | 20 | 0 | 0 | 100 | 52 | 25 | 0 | 10 | 25 |
| 1 | 0 | 15 | 0 | 0 | 0 | 12 | 15 | 10 | 0 | 0 |
| 4 | 0 | 0 | 50 | 0 | 100 | 68 | 40 | 67 | 70 | 25 |
| 5 | 0 | 10 | 25 | 0 | 0 | 40 | 45 | 45 | 40 | 25 |

| | | | | | | | | | | |
|---|---|---|---|---|---|---|---|---|---|---|
| 4 | 8.2 | 5 | 25 | 0 | 67 | 64 | 45 | 100 | 25 | 50 |
| 1 | 0 | 10 | 88 | 100 | 100 | 80 | 45 | 57 | 50 | 25 |
| 2 | 12 | 20 | 25 | 0 | 0 | 52 | 40 | 22 | 45 | 75 |
| 5 | 0 | 25 | 63 | 0 | 0 | 56 | 30 | 22 | 35 | 50 |
| 5 | 15 | 95 | 88 | 100 | 100 | 72 | 55 | 67 | 65 | 75 |
| 3 | 0 | 50 | 88 | 75 | 100 | 60 | 75 | 100 | 75 | 50 |
| 3 | 0 | 100 | 88 | 100 | 100 | 64 | 75 | 100 | 85 | 50 |
| 3 | 0 | 100 | 100 | 25 | 100 | 64 | 35 | 88 | 80 | 50 |
| 6 | 0 | 40 | 13 | 25 | 0 | 32 | 40 | 51 | 15 | 25 |
| 3 | 11.5 | 65 | 38 | 0 | 0 | 36 | 25 | 45 | 30 | 50 |
| 3 | 0 | 30 | 0 | 100 | 100 | 32 | 25 | | 15 | 0 |
| 2 | 0 | 0 | 50 | 0 | 100 | 60 | 75 | 45 | 60 | 25 |
| 3 | 15.6 | 95 | 88 | 75 | 100 | 64 | 55 | 100 | 80 | 50 |
| 3 | 26 | 75 | 50 | 50 | 100 | 64 | 50 | 57 | 55 | 25 |
| 3 | 0 | 25 | 50 | 0 | 0 | 48 | 45 | 43 | 40 | 25 |
| 6 | 0 | 50 | 100 | 25 | 100 | 52 | 45 | 57 | 45 | 25 |
| 3 | 18 | 100 | 100 | 100 | 100 | 80 | 80 | 100 | 90 | 50 |
| 3 | 24.5 | 95 | 88 | 100 | 100 | 68 | 75 | 67 | 75 | 50 |
| 5 | 8 | 80 | 88 | | 100 | 56 | 60 | 80 | 75 | 50 |
| 6 | 0 | 20 | 63 | 0 | 67 | 68 | 20 | 33 | 35 | 50 |
| 7 | 28.9 | 15 | 25 | 0 | 0 | 48 | 50 | 22 | 45 | 25 |
| 5 | 10.5 | 35 | 50 | 0 | 0 | 36 | 40 | 45 | 35 | 25 |
| 7 | 0 | 35 | 63 | 25 | 67 | 48 | 45 | 22 | 35 | 50 |
| 2 | 57.6 | 55 | 100 | | | 72 | 45 | 80 | 50 | 50 |
| 3 | 0 | 60 | 100 | 100 | 100 | 80 | 65 | 100 | 70 | 50 |
| 3 | 16.13 | 60 | 63 | 0 | 100 | 76 | 45 | 100 | 75 | 75 |
| 5 | 8.6 | 55 | 75 | 75 | 100 | 68 | 50 | 67 | 65 | 50 |
| 3 | 21.9 | 30 | 38 | 0 | 100 | 56 | 60 | 57 | 40 | 75 |
| 7 | 27 | 100 | 100 | 100 | 100 | 88 | 90 | 100 | 100 | 75 |
| 5 | 9 | 30 | 0 | 0 | 100 | 72 | 20 | 10 | 65 | 50 |
| 5 | 7 | 70 | 63 | 0 | 100 | 48 | 55 | 33 | 70 | 50 |
| 3 | 0 | 10 | 0 | 0 | 0 | 28 | 35 | 76 | 30 | 0 |
| 2 | 128.2 | 35 | 88 | 100 | 100 | 72 | 70 | 57 | 80 | 50 |
| 5 | 8 | 65 | 88 | 50 | 100 | 88 | 50 | 67 | 40 | 50 |
| 3 | 9.5 | 80 | 50 | 25 | 100 | 64 | 45 | 78 | 45 | 75 |
| 2 | 21.5 | 45 | 75 | 50 | 100 | 56 | 50 | 57 | 45 | 50 |
| 3 | 7 | 80 | 38 | 0 | 0 | 44 | 20 | 100 | 45 | 50 |
| 6 | 0 | 0 | 88 | 100 | 100 | 76 | 55 | 80 | 50 | 50 |
| 3 | 12.9 | 0 | 100 | 75 | 100 | 80 | 45 | 45 | 95 | 50 |
| 3 | 0 | 5 | 38 | 50 | 33 | 36 | 35 | 45 | 25 | 75 |
| 5 | 0 | 5 | 38 | 0 | 100 | 80 | 65 | | 50 | 50 |
| 2 | 0 | 70 | 63 | 0 | 33 | 60 | 50 | 67 | 50 | 25 |
| 5 | 0 | 80 | 63 | | 100 | 48 | 55 | 67 | 50 | 75 |
| 3 | 0 | 30 | 63 | 25 | 100 | 80 | 65 | 67 | 60 | 25 |
| 3 | 0 | 45 | 100 | 25 | | 80 | 70 | 45 | 65 | 50 |
| 5 | 0 | 80 | 75 | 75 | 100 | 84 | 75 | 78 | 70 | 75 |
| 5 | 9.9 | 80 | 100 | 50 | 100 | 80 | 65 | 57 | 50 | 25 |
| 5 | 14.25 | 80 | 63 | 0 | 100 | 80 | 55 | 45 | 70 | 50 |
| 5 | 45.4 | 65 | 100 | 100 | 100 | 72 | 50 | 59 | 55 | 25 |
| 7 | 25.8 | 5 | 25 | 25 | 67 | 64 | 50 | 57 | 70 | 75 |
| 6 | 4 | 15 | 63 | 25 | 100 | 72 | 60 | 78 | 95 | 25 |
| 5 | 82.5 | 100 | 100 | 100 | 100 | 80 | 65 | 90 | 65 | 100 |
| 7 | 48.75 | 55 | 88 | 75 | 100 | 76 | 75 | 90 | 50 | 75 |
| 4 | 0 | 10 | 75 | 0 | 0 | 84 | 40 | 78 | 35 | 25 |
| 5 | 38.95 | 95 | 100 | 100 | 100 | 76 | 65 | 90 | 90 | 100 |
| 5 | 20.08 | 60 | 25 | 0 | 0 | 48 | 45 | 45 | 65 | 75 |
| 3 | 8 | 55 | 50 | 25 | 100 | 64 | 25 | 100 | 60 | 50 |
| 3 | 0 | 65 | 63 | 50 | 100 | 84 | 70 | 67 | 60 | 25 |
| 7 | 65.4 | 95 | 63 | 25 | 33 | 72 | 70 | 100 | 75 | 50 |

| | | | | | | | | | | |
|---|---|---|---|---|---|---|---|---|---|---|
| 2 | 0 | 5 | 50 | 0 | 100 | 72 | 40 | 45 | 90 | 75 |
| 5 | 54 | 65 | 38 | 0 | 100 | 68 | 35 | 45 | 85 | 50 |
| 7 | 0 | 0 | 50 | | | 40 | 45 | 20 | | 50 |
| 7 | 26.95 | 45 | 100 | 50 | 100 | 80 | 50 | 67 | 50 | 50 |
| 5 | 54.5 | 75 | 100 | 100 | 100 | 84 | 85 | 67 | 90 | 75 |
| 7 | 0 | 30 | 100 | 75 | 100 | 88 | 55 | 57 | 25 | 75 |
| 5 | 33.6 | 70 | 100 | 50 | 100 | 80 | 65 | 57 | 90 | 75 |
| 7 | | 85 | 25 | 0 | 0 | 68 | 50 | 43 | 55 | 50 |
| 3 | | 10 | 38 | 25 | 33 | 64 | 50 | 22 | 40 | 75 |
| 7 | 15.6 | 95 | 100 | 100 | 100 | 84 | 90 | 100 | 70 | 50 |
| 3 | | 60 | 63 | 50 | 100 | 80 | 70 | 90 | 95 | 50 |
| 7 | 36 | 100 | 100 | 100 | 100 | 60 | 70 | 100 | 80 | 50 |
| 6 | | 35 | 88 | 100 | 100 | 80 | 40 | 100 | 50 | 25 |
| 3 | 51.2 | 50 | 75 | 50 | 100 | 88 | 45 | 100 | 85 | 75 |
| 3 | 8 | 50 | 100 | 100 | 100 | 80 | 75 | 90 | 70 | 75 |
| 5 | 12.95 | 45 | 38 | 0 | 100 | 56 | 25 | 22 | 30 | 25 |
| 3 | 12 | 60 | 63 | 25 | 67 | 52 | 40 | 88 | 45 | 50 |
| 5 | 17.2 | 50 | 88 | 50 | 100 | 76 | 50 | 67 | 45 | 50 |
| 5 | 53 | 55 | 50 | 0 | 100 | 60 | 55 | 55 | 35 | 75 |
| 6 | 0 | 0 | 25 | 0 | 0 | 36 | 15 | 22 | 40 | 0 |
| 2 | 0 | 15 | 25 | 0 | | 48 | 50 | 33 | 30 | 25 |
| 4 | 0 | 30 | 13 | 0 | | 24 | 15 | 0 | 10 | 0 |
| 5 | 30 | 80 | 100 | 75 | 100 | 72 | 55 | 80 | 55 | 75 |
| 5 | 14 | 15 | 13 | 0 | 100 | 64 | 45 | 0 | 20 | 25 |
| 5 | 15 | 75 | 63 | 0 | 0 | 56 | 55 | 35 | 45 | 50 |
| 7 | 11.5 | 70 | 0 | | 0 | 28 | 30 | 55 | 50 | 50 |
| 7 | 20 | 100 | 25 | | | 48 | 45 | 45 | 65 | 50 |
| 3 | 0 | 75 | 50 | 0 | 67 | 64 | 55 | 90 | 65 | 50 |
| 3 | 33.1 | 80 | 63 | 0 | 0 | 80 | 55 | 90 | 70 | 50 |
| 5 | 11.8 | 30 | 50 | 0 | 100 | 68 | 35 | 45 | 30 | 25 |
| 3 | 12 | 40 | 88 | 75 | 100 | 64 | 50 | 88 | 70 | 50 |
| 7 | 49 | 80 | 75 | 100 | 100 | 84 | 70 | 67 | 55 | 75 |
| 5 | 11.25 | 75 | 100 | 25 | 100 | 72 | 60 | 45 | 60 | 50 |
| 5 | 0 | 40 | 25 | 0 | 0 | 24 | 15 | 22 | 15 | 0 |
| 7 | 10.5 | 45 | 25 | 50 | 0 | 36 | 20 | 57 | 15 | 50 |
| 7 | 23.8 | 50 | 75 | 25 | 0 | 56 | 55 | 100 | 55 | 50 |
| 2 | 0 | 5 | 38 | 25 | 0 | 72 | 30 | 22 | 50 | 25 |
| 3 | 0 | 65 | 38 | 25 | 100 | 48 | 60 | 67 | 40 | 50 |
| 7 | 54.6 | 85 | 75 | 50 | 33 | 44 | 60 | 67 | 75 | 75 |
| 3 | 12 | 45 | 38 | 0 | 0 | 60 | 50 | 67 | 40 | 75 |
| 3 | 11.3 | 45 | 63 | 50 | 67 | 48 | 55 | 67 | 60 | 50 |
| 5 | 52.5 | 60 | 75 | 75 | 100 | 84 | 50 | 45 | 45 | 50 |
| 5 | 11.8 | 50 | 63 | 0 | 100 | 76 | 60 | 45 | 90 | 25 |
| 3 | 18 | 100 | 100 | 100 | 100 | 76 | 80 | 100 | 85 | 50 |
| 7 | 9.8 | 65 | 88 | 100 | 100 | 76 | 75 | 80 | 90 | 100 |
| 2 | 12.9 | 80 | 88 | 100 | 100 | 88 | 65 | 67 | 70 | 25 |
| 3 | 0 | 65 | 88 | 75 | 100 | 84 | 50 | 90 | 65 | 50 |
| 2 | 8 | 30 | 50 | 0 | 100 | 68 | 40 | 100 | 50 | 25 |
| 7 | 47 | 90 | 88 | 100 | 100 | 72 | 70 | 67 | 75 | 75 |
| 3 | 0 | 70 | 63 | 25 | 100 | 76 | 35 | 100 | 35 | 50 |
| 5 | 24.5 | 70 | 75 | 0 | 100 | 56 | 50 | 57 | 50 | 50 |
| 7 | 65.6 | 40 | 100 | 100 | 100 | 80 | 65 | 80 | 65 | 75 |
| 7 | 37.35 | 100 | 100 | 100 | 100 | 84 | 75 | 100 | 65 | 50 |
| 2 | 0 | 5 | 25 | 25 | 33 | 64 | 30 | 45 | 50 | 0 |
| 5 | 15.75 | | 100 | 75 | 100 | 72 | 50 | 57 | 80 | |
| 2 | 16 | 50 | 38 | 0 | 0 | 40 | 35 | 33 | 10 | 25 |
| 4 | 0 | 5 | 13 | 0 | 0 | 12 | 15 | 100 | 15 | 25 |
| 7 | 20 | 20 | 50 | | | 60 | 60 | 10 | 40 | 25 |
| 5 | 4.5 | 80 | 88 | 50 | 100 | 68 | 50 | 78 | 55 | 100 |

| | | | | | | | | | | |
|---|---|---|---|---|---|---|---|---|---|---|
| 3 | 17.2 | 80 | 75 | 25 | 100 | 80 | 60 | 80 | 50 | 25 |
| 5 | 0 | 65 | 75 | 0 | 100 | 84 | 65 | 57 | 50 | 100 |
| 4 | 52 | 95 | 88 | 75 | 100 | 60 | 55 | 90 | 40 | 50 |
| 3 | 0 | 55 | 100 | 25 | 100 | 72 | 60 | 57 | 20 | 50 |
| 1 | 0 | 20 | 0 | 100 | 0 | 16 | 25 | 80 | 15 | 25 |
| 1 | 10 | 55 | 100 | 100 | 100 | 72 | 75 | 80 | 65 | 75 |
| 3 | 15.3 | 75 | 63 | 75 | 67 | 48 | 45 | 67 | 45 | 75 |
| 5 | 3.5 | 100 | 100 | 100 | 100 | 88 | 70 | 100 | 100 | 50 |
| 6 | 18 | 95 | 88 | 100 | 100 | 76 | 75 | 100 | 55 | 50 |
| 3 | 0 | 45 | 75 | 75 | 33 | 76 | 70 | 100 | 60 | 25 |
| 3 | 0 | 65 | 100 | 75 | 100 | 88 | 70 | 90 | 65 | 50 |
| 2 | 17.2 | 10 | 63 | | | 72 | 60 | 78 | 60 | 75 |
| 5 | 14 | 60 | 75 | 50 | 100 | 68 | 70 | 67 | 80 | 50 |
| 7 | 0 | 60 | 88 | 0 | 100 | 76 | 45 | 67 | 45 | 50 |
| 6 | 0 | 75 | 75 | 100 | 100 | 88 | 75 | 100 | 80 | 50 |
| 5 | 20 | 15 | 25 | | 0 | 40 | 25 | 67 | 35 | 50 |
| 3 | 22.5 | 55 | 38 | 75 | 100 | 56 | 55 | 67 | 60 | 75 |
| 7 | 30.1 | 25 | 50 | 0 | 67 | 60 | 65 | 55 | 45 | 25 |
| 7 | 7.73 | 70 | 100 | 50 | 0 | 72 | 75 | 100 | 45 | 25 |
| 3 | 0 | 10 | 38 | 0 | 0 | 32 | 40 | 22 | 30 | 25 |
| 2 | 0 | 0 | 88 | | | 80 | 70 | 69 | 65 | 75 |
| 3 | 0 | 0 | 38 | 0 | 0 | 52 | 35 | 55 | 55 | 75 |
| 1 | 32.25 | 20 | 63 | 75 | 0 | 44 | 20 | 57 | 10 | 75 |
| 3 | 30.25 | 95 | 100 | 100 | 100 | 76 | 75 | 100 | 90 | 100 |
| 6 | 12 | 95 | 75 | 100 | 33 | | | 78 | 70 | 75 |
| 7 | 20.5 | 70 | 63 | 0 | 0 | 36 | 30 | 22 | 40 | 50 |
| 3 | 0 | 65 | 75 | 0 | 100 | 48 | 30 | 100 | 55 | 0 |
| 6 | 57.6 | 65 | 100 | 100 | 100 | 88 | 80 | 67 | 75 | 100 |
| 3 | 5.8 | 20 | 63 | 0 | 33 | 68 | 60 | 78 | 55 | 100 |
| 3 | 6 | 75 | 38 | 25 | 67 | 72 | 45 | 100 | 70 | 50 |
| 7 | 10.4 | 40 | 25 | 0 | 67 | 48 | 40 | 10 | 50 | 25 |
| 5 | 15.5 | 95 | 100 | 100 | 100 | 80 | 65 | 80 | 95 | 50 |
| 3 | 13.3 | 100 | 25 | 0 | | 68 | 65 | 100 | 45 | 75 |
| 3 | 17.2 | 30 | 13 | 0 | 0 | 24 | 30 | 67 | 25 | 50 |
| 3 | 49 | 80 | 38 | 0 | 0 | 68 | 50 | 100 | 40 | 25 |
| 6 | 15.2 | 50 | 88 | 75 | 100 | 84 | 70 | 57 | 60 | 75 |
| 5 | 0 | 65 | 75 | | | 48 | 35 | 67 | 35 | 25 |
| 3 | 24.5 | 60 | 63 | 75 | 33 | 56 | 50 | 100 | 80 | 50 |
| 3 | 16.1 | 40 | 13 | 0 | 33 | 76 | 45 | 57 | 60 | 25 |
| 7 | 0 | 20 | 88 | 0 | 67 | 64 | 45 | 20 | 20 | 50 |
| 6 | 0 | 25 | 38 | 0 | 0 | 60 | 60 | 55 | 90 | 75 |
| 5 | 31.35 | 95 | 100 | 75 | 33 | 64 | 75 | 80 | 75 | 100 |
| 5 | 24.28 | 85 | 75 | 100 | 100 | 68 | 70 | 78 | 65 | 100 |
| 2 | 28 | 35 | 88 | 100 | 100 | 68 | 60 | 100 | 75 | 50 |
| 6 | 0 | 0 | 63 | 25 | | 76 | 45 | 45 | 5 | 25 |
| 3 | 0 | 75 | 50 | 75 | 67 | 52 | 10 | 88 | 65 | 50 |
| 3 | 10.5 | 15 | 63 | 0 | 100 | 64 | 50 | 67 | 55 | 50 |
| 6 | 17.2 | 80 | 63 | | | 68 | 65 | 45 | 45 | 25 |
| 1 | 0 | 50 | 100 | 100 | 100 | 72 | 60 | 100 | 25 | 50 |
| 5 | 21 | 100 | 88 | 100 | 100 | 76 | 65 | 67 | 65 | 50 |
| 6 | 0 | 10 | 38 | | 100 | 60 | 45 | 67 | 55 | 25 |
| 3 | 21.5 | 45 | 75 | 50 | 100 | 48 | 50 | 100 | 90 | 50 |
| 3 | 0 | 65 | 50 | 0 | 100 | 84 | 60 | 90 | 45 | 25 |
| 6 | 12.25 | 15 | 50 | 25 | | 72 | 35 | 33 | 15 | 0 |
| 3 | 17.25 | 70 | 0 | 0 | 100 | 72 | 30 | 57 | 65 | 75 |
| 5 | 0 | 25 | 50 | 0 | 100 | 80 | 75 | 33 | 50 | 25 |
| 5 | 0 | 80 | 63 | | | 60 | 50 | 90 | 70 | 50 |
| 6 | 0 | 5 | 50 | 100 | 100 | 44 | 30 | 10 | 45 | 75 |
| 5 | 31.5 | 75 | 63 | 0 | 100 | 80 | 75 | 57 | 90 | 50 |

| | | | | | | | | | | |
|---|---|---|---|---|---|---|---|---|---|---|
| 3 | 78.5 | 85 | 100 | 50 | 100 | 72 | 55 | 67 | 70 | 75 |
| 2 | 0 | 95 | 63 | 0 | 67 | 76 | 55 | 100 | 45 | 50 |
| 2 | 0 | 0 | 50 | 0 | 0 | 48 | 45 | 67 | 55 | 50 |
| 2 | 10.75 | 5 | 75 | 0 | 33 | 48 | 65 | 67 | 55 | 50 |
| 7 | 0 | 50 | 25 | | | 60 | 50 | 22 | 35 | 25 |
| 5 | 33.25 | 80 | 88 | 75 | 100 | 68 | 45 | 67 | 70 | 100 |
| 2 | 20.3 | 30 | 63 | 25 | 100 | 68 | 70 | 45 | 80 | 50 |
| 3 | 28 | 35 | 63 | 50 | 33 | 40 | 25 | 55 | 60 | 25 |
| 5 | 0 | 30 | 50 | 0 | 33 | 60 | 20 | 45 | 35 | 25 |
| 5 | 17.5 | 55 | 75 | 25 | 100 | 72 | 55 | 57 | 70 | 50 |
| 3 | 14.1 | 60 | 75 | 75 | 100 | 80 | 65 | 45 | 35 | 25 |
| 4 | 0 | 0 | 100 | 100 | 100 | 80 | 60 | 80 | 70 | 50 |
| 3 | 10.5 | 80 | 75 | | 0 | 60 | 65 | 55 | 55 | 25 |
| 6 | 23.7 | 55 | 100 | 100 | 100 | 88 | 65 | 100 | 60 | 75 |
| 3 | 0 | 30 | 50 | 0 | 100 | 80 | 75 | 90 | 70 | 50 |
| 3 | 0 | 5 | 75 | 0 | 100 | 80 | 60 | 100 | 40 | 50 |
| 3 | 0 | 55 | 75 | 25 | 100 | 84 | 70 | 90 | 45 | 50 |
| 5 | 6 | 80 | 63 | 100 | 100 | 64 | 75 | 57 | 75 | |
| 3 | 0 | 95 | 63 | 100 | 33 | 76 | 55 | 90 | 75 | 50 |
| 3 | 37.8 | 65 | 75 | 75 | 100 | 72 | 50 | 78 | 60 | 75 |
| 3 | 11.45 | 30 | 75 | 100 | 100 | 60 | 50 | 88 | 65 | 50 |
| 3 | 22.8 | 40 | 100 | 100 | 100 | 84 | 80 | 69 | 85 | 50 |
| 5 | 0 | 50 | 63 | 100 | 33 | 80 | 50 | 67 | 60 | 50 |
| 7 | 0 | 20 | 100 | 0 | 100 | 80 | 55 | 57 | 75 | 75 |
| 3 | 14.5 | 90 | 50 | 0 | 0 | 64 | 65 | 100 | 55 | 50 |
| 3 | 0 | 30 | 0 | 0 | 0 | 44 | 35 | 10 | 50 | 50 |
| 2 | 0 | 30 | 0 | 0 | 0 | 24 | 15 | 0 | 15 | 75 |
| 3 | 26.2 | 30 | 63 | 0 | 0 | 68 | 35 | 35 | 60 | 50 |
| 3 | 0 | 0 | 50 | 50 | 33 | 40 | 35 | 45 | 40 | 50 |
| 5 | 27.5 | 65 | 13 | 0 | 100 | 76 | 15 | 33 | 40 | 25 |
| 3 | 0 | 60 | 100 | 100 | 100 | 40 | 40 | 67 | 50 | 75 |
| 5 | 22.7 | 95 | 100 | 100 | 100 | 88 | 90 | 100 | 90 | 100 |
| 3 | 0 | 40 | 38 | 25 | 0 | 48 | 35 | 33 | 75 | 50 |
| 5 | 27.5 | 35 | 0 | 0 | 0 | 48 | 35 | 0 | 90 | 25 |
| 6 | 10 | 65 | 100 | 100 | 100 | 72 | 60 | 90 | 60 | 50 |
| 3 | 21 | 90 | 25 | 0 | 0 | 56 | 40 | 45 | 65 | 50 |
| 5 | 0 | 60 | 63 | 0 | 100 | 60 | 40 | 55 | 50 | 50 |
| 3 | 29.5 | 80 | 88 | 50 | 100 | 60 | 60 | 100 | 75 | 50 |
| 5 | 32.2 | 80 | 100 | 100 | 100 | 72 | 75 | 67 | 50 | 100 |
| 2 | 19.3 | 25 | 50 | 0 | 100 | 60 | 45 | 45 | 20 | 25 |
| 5 | 5 | 65 | 38 | 100 | 100 | 48 | 25 | 45 | 60 | 50 |
| 7 | 14.36 | 20 | 50 | 25 | 100 | 80 | 60 | 22 | 65 | 50 |
| 5 | 12 | 70 | 88 | 100 | 100 | 88 | 65 | 57 | 65 | 75 |
| 5 | 30.05 | 75 | 100 | 100 | 100 | 84 | 85 | 78 | 65 | 75 |
| 2 | 0 | 5 | 75 | | 100 | 72 | 60 | 67 | 70 | 50 |
| 3 | 0 | 5 | 75 | 0 | 0 | 88 | 85 | 100 | 70 | 50 |
| 3 | 0 | 25 | 13 | 0 | 100 | 72 | 30 | 22 | 45 | 50 |
| 6 | 0 | 55 | 13 | 0 | 100 | 72 | 40 | 67 | 30 | 50 |
| 7 | 4.87 | 40 | 75 | 25 | 33 | 72 | 50 | 22 | 65 | 50 |
| 7 | 26 | 65 | 100 | 100 | 100 | 88 | 40 | 45 | 65 | 50 |
| 3 | 0 | 35 | 50 | 0 | 0 | 52 | 45 | 67 | 65 | 50 |
| 4 | 23.4 | 40 | 38 | 0 | 0 | 48 | 40 | 67 | 45 | 25 |
| 5 | 0 | 85 | 75 | 50 | 100 | 68 | 50 | 57 | 75 | 50 |
| 6 | 0 | 5 | 75 | 50 | 67 | 48 | 35 | 67 | 25 | 25 |
| 1 | 0 | 25 | 38 | 0 | 100 | 76 | 15 | 45 | 20 | 50 |
| 5 | 0 | 55 | 100 | 25 | 100 | 72 | 40 | 45 | 55 | 50 |
| 2 | 0 | 10 | 63 | 25 | 100 | 76 | 55 | 57 | 40 | 50 |
| 1 | 12 | 30 | 88 | 50 | 100 | 68 | 65 | 90 | 15 | 50 |
| 3 | 5.8 | 40 | 25 | 75 | 0 | 40 | 25 | | 30 | 25 |

| | | | | | | | | | | |
|---|---|---|---|---|---|---|---|---|---|---|
| 5 | 75 | 80 | 100 | 50 | 0 | 84 | 50 | 100 | 50 | 100 |
| 5 | 11.25 | 65 | 50 | 25 | 100 | 88 | 60 | 45 | 65 | 75 |
| 3 | 18 | 65 | 50 | 0 | 100 | 76 | 70 | 55 | | 50 |
| 3 | 0 | 95 | 63 | 0 | 0 | 68 | 75 | 100 | 75 | 50 |
| 2 | 36 | 20 | 50 | 0 | 0 | 60 | 55 | 22 | 60 | 50 |
| 2 | 0 | 15 | 88 | 50 | 100 | 60 | 35 | 69 | 70 | 75 |
| 5 | 0 | 60 | 63 | 0 | 0 | 44 | 40 | 33 | 30 | 50 |
| 5 | 0 | 50 | 50 | 0 | 0 | 48 | 40 | 45 | 50 | 25 |
| 5 | 25.6 | 95 | 63 | 50 | 67 | 56 | 60 | 67 | 70 | 75 |
| 5 | 0 | 10 | 38 | 25 | | 68 | 40 | 22 | | 25 |
| 3 | 24.9 | 100 | 50 | 0 | 0 | 68 | 65 | 76 | 55 | 100 |
| 3 | 80 | | 50 | 75 | 67 | 36 | 35 | 100 | 60 | 50 |
| 4 | 4 | 30 | 13 | 0 | 33 | 68 | 35 | 22 | 55 | 25 |
| 5 | 0 | 100 | 100 | 100 | 100 | 80 | 50 | 90 | 75 | 50 |
| 4 | 10.5 | 80 | 100 | 100 | 100 | 76 | 65 | 67 | 70 | 50 |
| 5 | 0 | 100 | 100 | 100 | 100 | 76 | 70 | 78 | 75 | 100 |
| 5 | 0 | 60 | 50 | 0 | 0 | 48 | 50 | 35 | 70 | 50 |
| 4 | 0 | 10 | 25 | 0 | 100 | 52 | 40 | 90 | 35 | 0 |
| 6 | 17.2 | 15 | 50 | 0 | 100 | 76 | 60 | 35 | 40 | 25 |
| 1 | 0 | 5 | 50 | | | 56 | 45 | 22 | 50 | 75 |
| 2 | 0 | 5 | 100 | | | 84 | 80 | 60 | 50 | 50 |
| 4 | 19.8 | 75 | 75 | 50 | 100 | 68 | 65 | 67 | 70 | 25 |
| 5 | 11.6 | 85 | 88 | 100 | 100 | 84 | 70 | 78 | 65 | 50 |
| 3 | 4 | 15 | 38 | 0 | 0 | 56 | 25 | 67 | 15 | 25 |
| 5 | 0 | 45 | 63 | 75 | 33 | 72 | 45 | 22 | 50 | 25 |
| 5 | 22.1 | 85 | 63 | 75 | 100 | 52 | 45 | 67 | 75 | 50 |
| 5 | 11.5 | 50 | 38 | 0 | 100 | 56 | 45 | 22 | 25 | 25 |
| 5 | 0 | 85 | 88 | 75 | 100 | 52 | 45 | 67 | 35 | 25 |
| 5 | 0 | 85 | 88 | 100 | 100 | 84 | 55 | 57 | 60 | 50 |
| 7 | 79.85 | | 50 | | 100 | 52 | 50 | 20 | 65 | 50 |
| 3 | 38 | 100 | 100 | 100 | 100 | 84 | 85 | 100 | | 50 |
| 3 | 17.3 | 60 | 75 | 50 | 100 | 68 | 45 | 100 | 75 | 75 |
| 4 | 17.8 | 100 | 100 | 100 | 100 | 84 | 85 | 100 | 95 | 100 |
| 3 | 29 | 5 | 75 | 0 | 0 | 52 | 30 | 57 | 40 | 50 |
| 7 | 27 | 90 | 63 | 25 | 100 | 60 | 65 | 90 | 70 | 50 |
| 5 | 85 | 90 | 63 | 50 | 100 | 84 | 70 | 45 | 55 | 75 |
| 7 | 0 | 20 | 63 | 0 | 100 | 72 | 65 | 45 | 60 | 50 |
| 3 | 0 | 40 | 13 | 0 | 0 | 28 | 15 | 100 | 40 | 50 |
| 4 | 4.3 | 0 | 63 | 0 | 100 | 68 | 55 | 22 | 35 | 25 |
| 5 | 43 | 80 | 38 | 25 | 0 | 72 | 40 | 90 | 65 | 100 |
| 5 | 69 | 80 | 50 | 100 | 0 | 56 | 45 | 57 | 65 | 50 |
| 3 | 17.6 | 95 | 63 | 100 | 67 | 48 | 40 | 78 | 90 | 50 |
| 3 | 47 | 90 | 63 | 100 | 100 | 76 | 70 | 90 | 75 | 50 |
| 5 | 0 | 60 | 88 | 50 | 33 | 76 | 40 | 67 | 55 | 25 |
| 5 | 96 | 95 | 100 | 100 | 100 | 80 | 70 | 90 | 85 | 100 |
| 3 | 20.5 | 100 | 75 | 100 | | 52 | 60 | 100 | 70 | 50 |
| 4 | 0 | 10 | 63 | 25 | 100 | 68 | 40 | 78 | 15 | 50 |
| 3 | 41.1 | 90 | 100 | 100 | 100 | 80 | 70 | 100 | 95 | 75 |
| 5 | 19.3 | 70 | 100 | 25 | 100 | 76 | 60 | 45 | 45 | 50 |
| 5 | 0 | 5 | 38 | 0 | 0 | 48 | 25 | 35 | 0 | 0 |
| 3 | 11.35 | 25 | 63 | 0 | 100 | 68 | 65 | 100 | 55 | 25 |
| 1 | 38.5 | 75 | 100 | 100 | 100 | 72 | 90 | 100 | 80 | 50 |
| 5 | 43.6 | 65 | 100 | 100 | 100 | 68 | 65 | 100 | 80 | 50 |
| 6 | 35.8 | 65 | 63 | 0 | 0 | 52 | 45 | 67 | 40 | 50 |
| 3 | 10.5 | 95 | 50 | 0 | 67 | 68 | 55 | 100 | 35 | 25 |
| 5 | 82.6 | 45 | 0 | 0 | 100 | 76 | 65 | 33 | 45 | 25 |
| 2 | 98 | 90 | 88 | 100 | 100 | 88 | 85 | 90 | 70 | 75 |
| 3 | 20 | 55 | 88 | 25 | | | | 67 | 65 | 50 |
| 7 | 53 | 65 | 100 | 75 | 100 | 80 | 75 | 90 | 85 | 50 |

| | | | | | | | | | | |
|---|---|---|---|---|---|---|---|---|---|---|
| 5 | 18.5 | 90 | 100 | 100 | 100 | 76 | 70 | 90 | 65 | 75 |
| 3 | 35 | 100 | 100 | 100 | 100 | 76 | 80 | 100 | 95 | 50 |
| 5 | 23.05 | 90 | 100 | 100 | 100 | 68 | 65 | 80 | 80 | 75 |
| 3 | 0 | 10 | 50 | 0 | 100 | 72 | 40 | 45 | 30 | 25 |
| 4 | 0 | 5 | 38 | 0 | 33 | 60 | 15 | 45 | 25 | 25 |
| 3 | 7.5 | 85 | 75 | 50 | 100 | 80 | 55 | 45 | 60 | 50 |
| 3 | 7.5 | 100 | 88 | 100 | 100 | 84 | 65 | 100 | 90 | 50 |
| 3 | 38.6 | 90 | 63 | 100 | 100 | 84 | 75 | 45 | 40 | 50 |
| 5 | 18.1 | 85 | 100 | 75 | 100 | 80 | 70 | 67 | 70 | 100 |
| 7 | 0 | 35 | 75 | 25 | 100 | 88 | 55 | 100 | 45 | 100 |
| 3 | 8.6 | 10 | 88 | 0 | 100 | 40 | 35 | 100 | 80 | 50 |
| 3 | 0 | 30 | 50 | | 100 | 68 | 25 | 78 | 55 | 50 |
| 3 | 0 | 80 | 75 | 0 | 100 | 88 | 55 | 67 | 70 | 75 |
| 2 | 26.8 | 30 | 50 | | 0 | 48 | 55 | 22 | 50 | 75 |
| 2 | 12.9 | 0 | 100 | 100 | 100 | 72 | 70 | 55 | 70 | 50 |
| 7 | 12 | 95 | 88 | 100 | 0 | 88 | 85 | 100 | 70 | 50 |
| 3 | 17.5 | 35 | 75 | 0 | 0 | 64 | 50 | 78 | 90 | 75 |
| 3 | 0 | 75 | 88 | 100 | 100 | 80 | 60 | 100 | 60 | 50 |
| 3 | 0 | 95 | 75 | 100 | 100 | 76 | 55 | 100 | 60 | 50 |
| 2 | 14 | 25 | 63 | 100 | 67 | 68 | 60 | 67 | 55 | 50 |
| 3 | 16.6 | 100 | 75 | 100 | 100 | 80 | 55 | 100 | 80 | 50 |
| 4 | 6.45 | 0 | 50 | 0 | 0 | 52 | 15 | 0 | 20 | 0 |
| 1 | 15.6 | 20 | 38 | 75 | 33 | 44 | 50 | 67 | 60 | 75 |
| 7 | 0 | 65 | 38 | 0 | 33 | 60 | 30 | 67 | 45 | 25 |
| 7 | 9.5 | 50 | 88 | 25 | 100 | 68 | 30 | 100 | 35 | 50 |
| 5 | 72 | 100 | 100 | 100 | 100 | 88 | 90 | 100 | 95 | 100 |
| 3 | 0 | 10 | 63 | 25 | 33 | 64 | 45 | 45 | 30 | 50 |
| 5 | 20.25 | 90 | 88 | 75 | 100 | 76 | 55 | 57 | 75 | 75 |
| 3 | 27.5 | 90 | 75 | 75 | 100 | 64 | 60 | 100 | 70 | 75 |
| 1 | 0 | 35 | 63 | 75 | 67 | 56 | 40 | 67 | 30 | 50 |
| 7 | 37.95 | 70 | 63 | 0 | 100 | 56 | 60 | 45 | 40 | 50 |
| 3 | 0 | 85 | 75 | 100 | 100 | 68 | 40 | 90 | 100 | 50 |
| 3 | 0 | 10 | 75 | 0 | 67 | 56 | 45 | 67 | 45 | 25 |
| 6 | 0 | 0 | 100 | | 100 | 84 | 45 | 100 | 55 | 0 |
| 3 | 60 | 100 | 100 | 100 | 100 | 80 | 80 | 100 | 90 | 50 |
| 3 | 0 | 95 | 75 | 25 | 33 | 72 | 45 | 100 | 50 | 50 |
| 6 | 12 | 15 | 38 | 0 | 0 | 60 | 40 | 22 | 35 | 0 |
| 3 | 0 | 5 | 0 | 0 | 0 | 36 | 10 | 100 | 10 | 0 |
| 3 | 0 | 0 | 63 | 0 | 0 | 68 | 50 | 33 | 30 | 50 |
| 3 | 0 | 30 | 75 | 25 | 100 | 64 | 60 | 100 | 90 | 50 |
| 3 | 0 | 20 | 50 | 0 | 0 | 64 | 80 | 31 | 60 | 50 |
| 5 | 10.5 | 70 | 63 | 25 | 0 | 40 | 40 | 45 | 25 | 0 |
| 3 | 41.05 | 90 | 100 | 100 | 100 | 88 | 65 | 100 | 70 | 50 |
| 5 | 0 | 20 | 25 | 0 | 67 | 56 | 35 | 10 | 55 | 50 |
| 6 | 0 | 40 | 25 | 0 | 0 | 52 | 35 | 33 | 35 | 0 |
| 5 | 0 | 75 | 75 | 0 | 100 | 72 | 55 | 57 | 65 | 50 |
| 3 | 16 | 50 | 100 | 50 | 67 | 64 | 35 | 35 | 35 | 50 |
| 3 | 24.7 | 80 | 88 | 0 | 100 | 72 | 70 | 67 | 70 | 75 |
| 6 | 7.6 | 70 | 100 | | | 76 | 40 | 60 | 40 | 75 |
| 5 | 29.4 | 100 | 88 | 100 | 100 | 56 | 60 | 90 | 75 | 75 |
| 2 | 12 | 30 | 88 | 100 | 100 | 72 | 60 | 100 | 75 | 50 |
| 5 | 10.5 | 45 | 63 | 0 | 100 | 52 | 60 | 45 | 65 | 25 |
| 3 | 0 | 90 | 75 | | 67 | 76 | 65 | 100 | 70 | 50 |
| 3 | 0 | 5 | 63 | 0 | 100 | 76 | 75 | 100 | 90 | 50 |
| 7 | 0 | 0 | 50 | | | | | | 50 | 50 |
| 5 | 8.5 | 70 | 75 | 25 | 100 | 68 | 30 | 45 | 45 | 25 |
| 6 | 43.1 | 80 | 50 | 0 | 0 | 36 | 40 | 78 | 45 | 25 |
| 4 | 0 | 15 | 50 | 50 | 100 | 60 | 60 | 57 | 45 | 25 |
| 6 | 17.25 | 35 | 100 | 100 | 100 | 76 | 65 | 67 | 55 | 50 |

| | | | | | | | | | | |
|---|---|---|---|---|---|---|---|---|---|---|
| 5 | 47.5 | 100 | 100 | 100 | 100 | 80 | 70 | 100 | 100 | 75 |
| 6 | 0 | 45 | 88 | 50 | 67 | 64 | 40 | 100 | 55 | 75 |
| 7 | 5.8 | 35 | 38 | 25 | 0 | 60 | 50 | 33 | 10 | 0 |
| 4 | 0 | 0 | 13 | 0 | 0 | 48 | 20 | 22 | 0 | 0 |
| 3 | 9.8 | 55 | 75 | 25 | 100 | 88 | 70 | 78 | 40 | 75 |
| 3 | 30.1 | 0 | 38 | | | 40 | 35 | 45 | 45 | 25 |
| 3 | 5.75 | 55 | 100 | 100 | 100 | 56 | 35 | 67 | 65 | 50 |
| 6 | 24.9 | 85 | 88 | 75 | 100 | 68 | 60 | 90 | 55 | 75 |
| 3 | 0 | 0 | 50 | 25 | 67 | 56 | 65 | 57 | 85 | 75 |
| 3 | 0 | 65 | 50 | 0 | 67 | 56 | 50 | 100 | 55 | 75 |
| 5 | 35.5 | 80 | 75 | 100 | 67 | 60 | 65 | 90 | 60 | 50 |
| 5 | 0 | 60 | 25 | 50 | | 28 | 20 | 45 | 50 | 0 |
| 5 | 0 | 20 | 63 | 0 | 100 | 72 | 60 | 22 | 30 | 50 |
| 3 | 0 | 20 | 0 | 75 | 67 | 28 | 25 | 53 | 35 | 50 |
| 3 | 0 | 10 | 50 | 100 | 100 | 52 | 50 | 76 | 70 | 50 |
| 3 | 0 | 55 | 75 | 50 | 0 | 68 | 55 | 78 | 40 | 75 |
| 5 | 10.3 | 60 | 63 | 0 | 100 | 72 | 50 | 35 | 40 | 50 |
| 5 | 12.35 | 85 | 100 | 100 | 100 | 64 | 55 | 67 | 60 | 75 |
| 3 | 0 | 15 | 50 | 0 | 0 | 64 | 50 | 100 | 35 | 25 |
| 5 | 18 | 60 | 63 | 25 | 33 | 52 | 40 | 57 | 40 | 25 |
| 1 | 81 | 30 | 25 | 0 | 67 | 68 | 70 | 12 | 65 | 25 |
| 5 | 33.5 | 90 | 63 | 75 | 67 | 64 | 65 | 45 | 35 | 50 |
| 5 | 6 | 80 | 100 | 100 | 100 | 64 | | 78 | 50 | 50 |
| 3 | 0 | 0 | 25 | 0 | 0 | 52 | 65 | 57 | 50 | 100 |
| 3 | 6 | 75 | 63 | 0 | 0 | 72 | 50 | 22 | 20 | 25 |
| 5 | 3.5 | 70 | 63 | 0 | 100 | 76 | 55 | 57 | 70 | 100 |
| 3 | 0 | 45 | 75 | 50 | 100 | 64 | 55 | 67 | 45 | 50 |
| 5 | 0 | 65 | 63 | 0 | 0 | 48 | 40 | 55 | 50 | 50 |
| 5 | 9.4 | 35 | 63 | 25 | 100 | 84 | 60 | 22 | 90 | 50 |
| 3 | 0 | 70 | 50 | 0 | 100 | 60 | 70 | 45 | 70 | 50 |
| 4 | 0 | 10 | 50 | | | 84 | 30 | 100 | 30 | 25 |
| 3 | 0 | 40 | 88 | 0 | 100 | 76 | 85 | 67 | 70 | 25 |
| 3 | 0 | 45 | 88 | 50 | 100 | 72 | 60 | 78 | 70 | 50 |
| 7 | 13.3 | 85 | 63 | 0 | 100 | 76 | 55 | 45 | 55 | 25 |
| 7 | 0 | 90 | 50 | 0 | 0 | 56 | 45 | 33 | 25 | 50 |
| 7 | 0 | 25 | 100 | 100 | 100 | 88 | 70 | 47 | 60 | 75 |
| 3 | 0 | 5 | 13 | 100 | 0 | 40 | 20 | 33 | 55 | 25 |
| 3 | 30.4 | 20 | 88 | 0 | 100 | 76 | 55 | 100 | 30 | 25 |
| 3 | 0 | 10 | 13 | 25 | 0 | 36 | 50 | 22 | 5 | 50 |
| 4 | 6.25 | 80 | 88 | 75 | 100 | 64 | 55 | 67 | 50 | 50 |
| 5 | 15.6 | 75 | 100 | 75 | 100 | 80 | 60 | 78 | 100 | 50 |
| 3 | 7.5 | 95 | 88 | 100 | 100 | 64 | 65 | 100 | 75 | 50 |
| 3 | 0 | 40 | 75 | 25 | | 68 | 50 | 100 | 65 | 50 |
| 3 | 39.6 | 85 | 88 | 100 | 100 | 64 | 75 | 78 | 75 | 75 |
| 4 | 27.25 | 85 | 50 | 0 | 67 | 68 | 25 | 100 | 40 | 25 |
| 7 | 0 | 10 | 25 | 0 | 0 | 76 | 55 | 10 | 40 | 0 |

Diagnosis: 1 = amputation; 2 = SCI; 3 = Brain injury; 4 = MS; 5 = Chronic pain; 6 = Neuro other; 7 = Other
Movement intensity: Activity specific intensity (MET) * hrs/week.
